# Photocatalytic Oxidative Desulfurization of H_2_S: A Review of Photocatalysts, Reaction Mechanisms, and Future Perspectives

**DOI:** 10.3390/ijms27219693

**Published:** 2026-10-29

**Authors:** Minghui Zhou, Hanyu Kong, Shiyi Wang, Mahmoud Samy, Shaojun Yuan

**Affiliations:** 1 Low-Carbon Technology & Chemical Reaction Engineering Lab, College of Chemical Engineering, Sichuan University, Chengdu 610065, China; zmh2173405700@163.com (M.Z.); shiyiwangg@gmail.com (S.W.); 2 College of Electronics and Information Engineering, Sichuan University, Chengdu 610065, China; 13915053658@163.com; 3 Public Works Engineering Department, Faculty of Engineering, Mansoura University, Mansoura 35516, Egypt; mahmoud.samy@ejust.edu.eg

**Keywords:** hydrogen sulfide, photocatalytic oxidation, photocatalysts, reaction mechanism, catalyst deactivation

## Abstract

Hydrogen sulfide (H_2_S) is a highly toxic, corrosive, and malodorous sulfur-containing pollutant emitted from diverse industrial and agricultural activities. Its low concentration, dispersed distribution, and intermittent emission characteristics often limit the effectiveness and economic feasibility of conventional desulfurization technologies. Photocatalytic desulfurization has emerged as a promising approach for H_2_S removal owing to its ability to utilize light energy under ambient conditions without generating secondary pollution. Among the various photocatalysts investigated, TiO_2_ remains the most extensively studied material. To improve photocatalytic performance, strategies such as elemental doping, defect engineering, heterojunction construction, and support coupling have been widely adopted to enhance visible-light absorption, promote charge separation, and improve resistance to deactivation. In addition, emerging photocatalysts, including metal–organic frameworks (MOFs), graphitic carbon nitride (g-C_3_N_4_), conjugated polymers, and carbon-based composites, have shown considerable potential for improving H_2_S adsorption, activation, and visible-light utilization. Photocatalytic H_2_S oxidation generally proceeds via sulfate or elemental sulfur formation pathways; however, both can result in sulfur accumulation on catalyst surfaces, leading to performance deterioration. Consequently, developing photocatalytic systems with efficient solar-light utilization, high sulfur tolerance, and long-term operational stability remains a critical challenge. This review summarizes recent advances in photocatalytic H_2_S oxidation, focusing on photocatalyst design, H_2_S removal performance, reaction mechanisms, and catalyst deactivation behaviors. Future challenges and research directions for the development of efficient and durable photocatalytic desulfurization technologies are also discussed.

## 1. Introduction

Hydrogen sulfide (H_2_S) is a gaseous pollutant characterized by an offensive odor, high toxicity, and strong corrosiveness, making its emissions a long-standing concern in the fields of industrial hygiene and environmental health [1]. Substantial quantities of H_2_S are released worldwide from a variety of anthropogenic activities [2], including natural gas extraction and purification, petroleum refining [3], biogas production [4], and livestock farming [5,6,7], as well as from natural processes such as volcanic eruptions, oceanic emissions, and volatilization from subtropical forest soils [8]. Global annual H_2_S emissions have been estimated at approximately 7.72 million tons [9]. H_2_S possesses an extremely low odor threshold of approximately 0.5 ppb. The Occupational Safety and Health Administration (OSHA) has established a workplace exposure limit of 20 ppm, whereas exposure to concentrations as high as 700 ppm can cause rapid fatality [10]. Therefore, the development of substantial H_2_S control technologies is substantial for practical applications [11,12].

Conventional H_2_S removal technologies, including the Claus process, wet and dry desulfurization, and chemical oxidation, have been extensively employed for industrial desulfurization [13,14,15,16,17,18,19,20]. However, these approaches generally suffer from high energy consumption, secondary pollutant generation, equipment corrosion, and limited effectiveness in treating dilute H_2_S streams [15,16,17,18,19,20,21,22]. Similarly, thermocatalytic oxidation is constrained by catalyst deactivation, limited catalyst durability, and inadequate product selectivity [23,24,25,26,27]. Consequently, there is an urgent need to develop energy-efficient and environmentally sustainable technologies capable of effectively removing dilute and dispersed H_2_S emissions.

**Figure 1:**
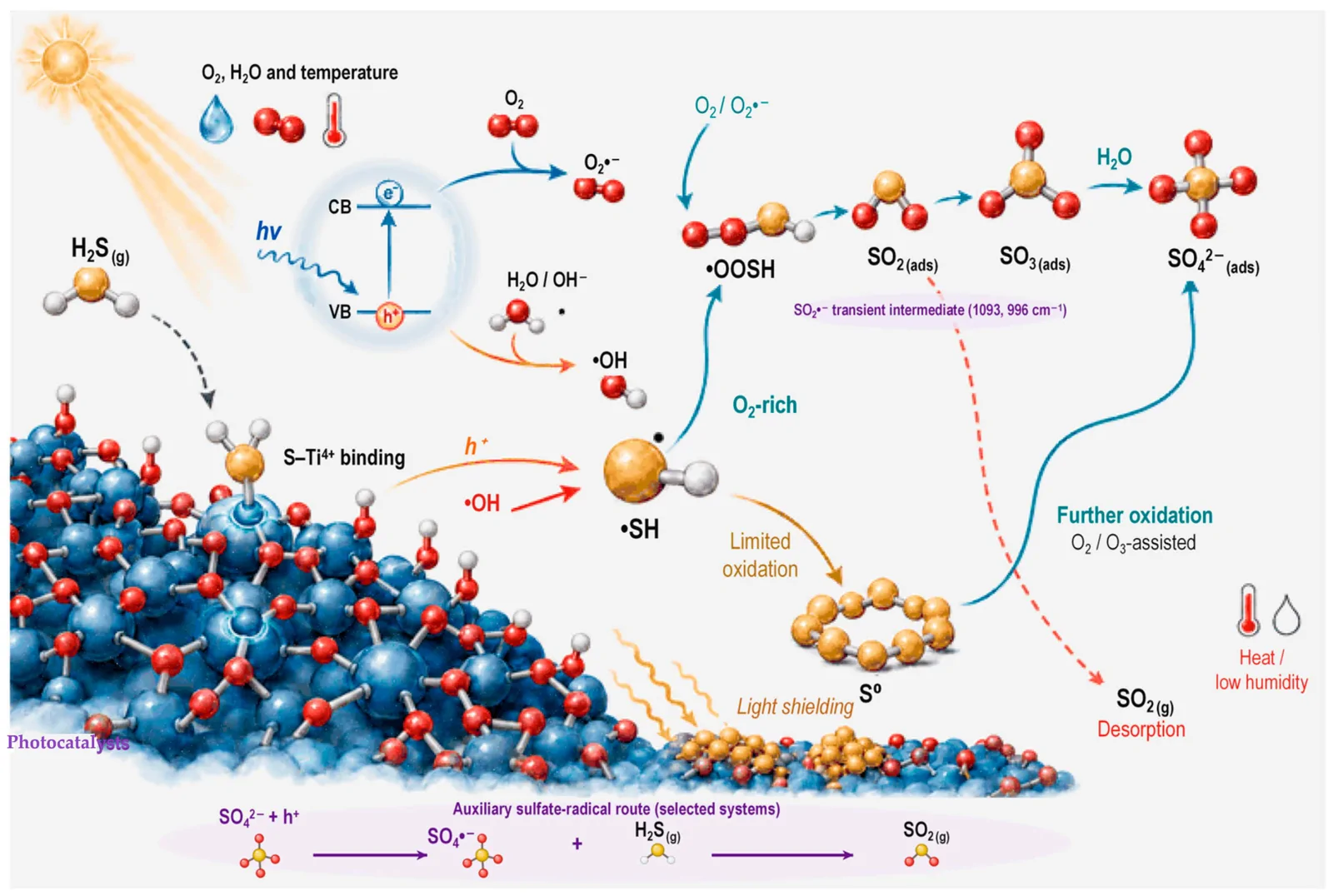
Reaction pathways for photocatalytic H_2_S oxidation on photocatalysts under different reaction conditions.

Among emerging treatment technologies, photocatalytic oxidation (PCO) has attracted considerable attention because it can utilize light energy to drive chemical reactions under ambient temperature and pressure conditions, while offering simple process operation and eliminating the need for additional chemical reagents. In H_2_S treatment, photocatalytic processes generally proceed through two main pathways: (i) photocatalytic decomposition, which converts H_2_S into hydrogen and elemental sulfur (H_2_S → H_2_ + S) [28,29,30,31], and (ii) photocatalytic oxidation, which transforms H_2_S into sulfate species (H_2_S → SO_4_^2−^) [32]. Compared with the photocatalytic decomposition, photocatalytic oxidation is more suitable for air purification [33] and the control of dispersed emission sources [5,34], as it avoids the safety risks and product separation challenges associated with hydrogen generation (Figure 1).

From a thermodynamic perspective, H_2_S is favorable substrate for photocatalytic conversion. The minimum dissociation energy required for its decomposition into hydrogen and sulfur is approximately 0.2 eV per molecule, which is substantially lower than that required for water splitting. Despite this advantage, practical applications remain constrained by thermodynamic limitations and relatively low single-pass conversion efficiencies [3,35]. Two major challenges currently hinder the large-scale deployment of H_2_S photocatalytic systems. First, in liquid-phase systems, the accumulation of polysulfide ions (S_n_^2−^) and sulfur deposition can hinder light penetration and cause photocatalyst deactivation [36]. Second, the rapid recombination of photogenerated charge carriers significantly decreases quantum efficiency [37], thereby necessitating larger reactor volumes to achieve the desired conversion performance. As a result, reactor construction costs may account for as much as 95% of the total capital investment [38].

Previous studies have demonstrated that in the presence of dissolved oxygen and under UVC irradiation, H_2_S conversion efficiencies exceeding 80% can be achieved at initial H_2_S concentrations of 5–14 mg L^−1^ and residence time of less than 5 min. Under these conditions, the quantum efficiency can reach 70%, and the reaction rate is approximately 1650 times greater than that of the corresponding dark oxidation process [39]. Compared with other H_2_S treatment technologies, including direct photocatalytic decomposition [40], electron-beam radiolysis [41], photoelectrochemical systems [42,43,44], plasma technologies [45], and advanced oxidation processes such as Fenton-based oxidation [3,17,46,47], photocatalytic oxidation offers notable advantages. These include a simple process flow, ease of miniaturization, and operation without the need for external chemical reagents. Moreover, the process can be powered directly by solar irradiation or energy-efficient LED light sources. These advantages highlight its potential for treating low-concentration and geographically dispersed H_2_S emissions from sources such as livestock farms and wastewater treatment facilities.

TiO_2_ remains the most extensively studied photocatalyst owing to its excellent chemical stability, low cost, and environmental benignity. However, its practical application is hindered by several intrinsic limitations, including a wide band gap (~3.2 eV), poor visible-light utilization, rapid electron–hole recombination, and sulfur-induced catalyst deactivation [48]. Although regeneration strategies, such as alkaline washing, can partially restore catalytic activity and adsorption capacity [49], the inherently weak interaction between H_2_S molecules and TiO_2_ surfaces continues to limit H_2_S adsorption and activation, thereby constraining the overall photocatalytic performance [50,51].

To overcome the limitations of conventional photocatalytic systems, various catalyst modification strategies have been developed, including metal and non-metal doping [52], heterojunction construction, morphology engineering, and defect engineering [53]. These approaches aim to enhance visible-light absorption, promote photogenerated charge separation, and improve long-term operational stability. Concurrently, advances in photocatalytic reactor design, such as annular reactors, packed-bed reactors, and honeycomb-supported configurations [54,55], combined with computational fluid dynamics (CFD) simulations [34], have provided crucial guidance for process optimization and scale-up.

In addition to photocatalyst engineering and reactor design, emerging photocatalytic materials have opened new avenues for efficient H_2_S oxidation. Photocatalysts based on metal–organic frameworks (MOFs) [56,57] and donor–acceptor heterostructure systems [58] have attracted increasing attention due to their enhanced visible-light absorption, superior charge separation, and high ROS generation efficiency. These advanced material platforms offer promising opportunities for developing highly efficient, stable, and sustainable photocatalytic technologies for H_2_S abatement.

In practical applications, H_2_S emissions are typically characterized by low concentrations, intermittent release patterns, and coexistence with complex gas mixtures, posing remarkable challenges for photocatalytic oxidation. For example, Lee et al. investigated a UV-A (365 nm)/TiO_2_ photocatalytic system under simulated poultry-house conditions containing low concentrations of mixed gaseous pollutants [59]. The study found that H_2_S removal was negligible, whereas NH_3_, N_2_O, and O_3_ were removed to varying extents, highlighting differences in photocatalytic reactivity and selectivity among pollutants [59,60]. Similarly, Ma et al. compared the photocatalytic hydrogen production performance of TiO_2_, CdS, and ZnS during H_2_S decomposition under anaerobic gas–solid conditions. CdS exhibited negligible activity in the gas phase (<0.5 μmol h^−1^), but significantly higher activity in liquid-phase systems [61], underscoring the critical influence of reaction phase. Such discrepancies likely arise from the presence of hole scavengers in liquid systems (e.g., sulfite ions, SO_3_^2−^), which enhance charge separation and suppress electron–hole recombination, as well as differences in surface reaction pathways and reactant adsorption behavior between phases. Despite these advances, several challenges continue to impede practical deployment of photocatalytic H_2_S removal technologies. These include competitive adsorption at active sites, catalyst poisoning, and difficulties in separating and managing reaction products. As a result, photocatalytic oxidation for H_2_S abatement remains at approximately Technology Readiness Level (TRL) 3, corresponding to laboratory-scale proof-of-concept validation, and considerable barriers to industrial implementation persist [62].

**Figure 2:**
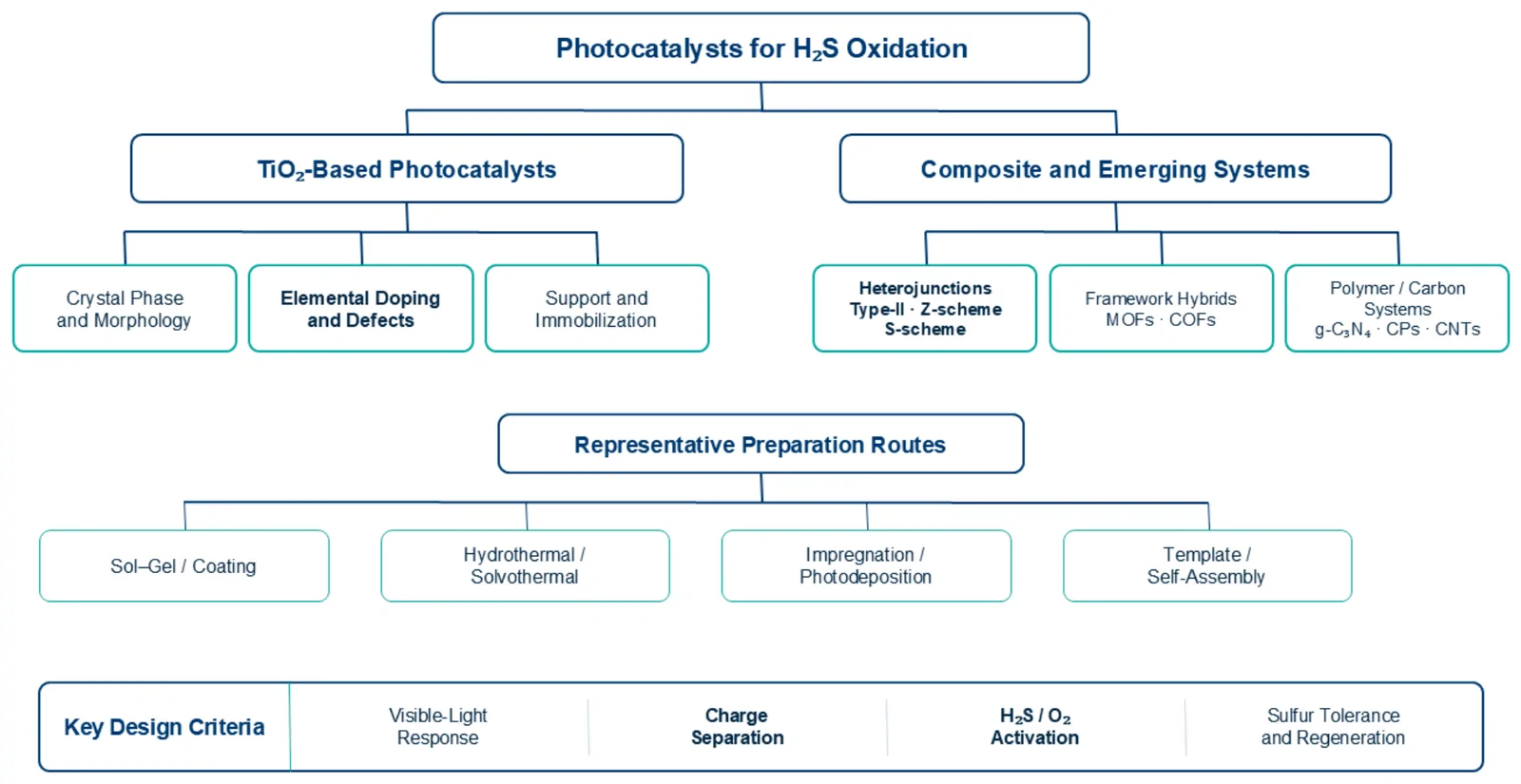
Catalyst types, preparation methods, and design criteria for photocatalytic H_2_S removal.

Therefore, this review systematically summarizes recent advances in TiO_2_-based and TiO_2_-derived composite photocatalysts for photocatalytic H_2_S oxidation, with particular emphasis on photocatalyst design, reaction mechanisms, reactor engineering, catalyst deactivation and regeneration (Figure 2), and the key challenges and future directions toward practical implementation. By critically assessing the current state of the field and identifying remaining knowledge gaps, these efforts are expected to facilitate the rational design of high-performance photocatalytic systems and accelerate the translation of photocatalytic H_2_S oxidation from laboratory-scale research to real-world environmental applications, ultimately providing energy-efficient, environmentally sustainable, and economically viable solutions for H_2_S emission control.

## 2. Advance in Photocatalysts for the Photocatalytic Oxidation of H_2_S

### 2.1. TiO_2_-Based Photocatalysts

Anatase and rutile are the two most common and extensively studied crystalline phases of TiO_2_. Compared with rutile, anatase has a wider band gap (~3.2 eV) and a more negative conduction band potential, enabling photogenerated electrons to exhibit stronger reducing power. Anatase also generally possesses a higher specific surface area and a greater concentration of surface oxygen vacancies, both of which facilitate reactant adsorption and enhance charge-carrier separation and migration. By contrast, rutile exhibits a narrower band gap (~3.0 eV), resulting in a broader light-response range, while its higher crystallinity and superior carrier mobility are beneficial for charge transport. Notably, mixed-phase anatase/rutile systems, such as the commercially available P25 photocatalyst (anatase/rutile mass ratio ≈ 80:20), often display higher photocatalytic activity than either pure phase. This enhancement is primarily attributed to the formation of a surface phase junction at the anatase-rutile interface, which promotes spatial separation of photogenerated electron–hole pairs and suppresses charge recombination [63]. In-doped TiO_2_ photocatalytic systems, photogenerated electrons generally exist in two forms. The first comprises accumulated electrons located in the conduction band or shallow trap states, which exhibit high mobility and strong reducing power, enabling direct participation in multielectron transfer reactions during H_2_S oxidation. The second consists of electrons trapped in intermediate or deep energy states. Owing to their lower reactivity, these trapped electrons can be effectively utilized only in the presence of electron acceptors with sufficiently positive electron affinity or favorable reduction potentials [64]. Therefore, regulating charge-carrier generation, separation, transport, and utilization has become a critical strategy for enhancing the photocatalytic performance of TiO_2_.

**Figure 3:**
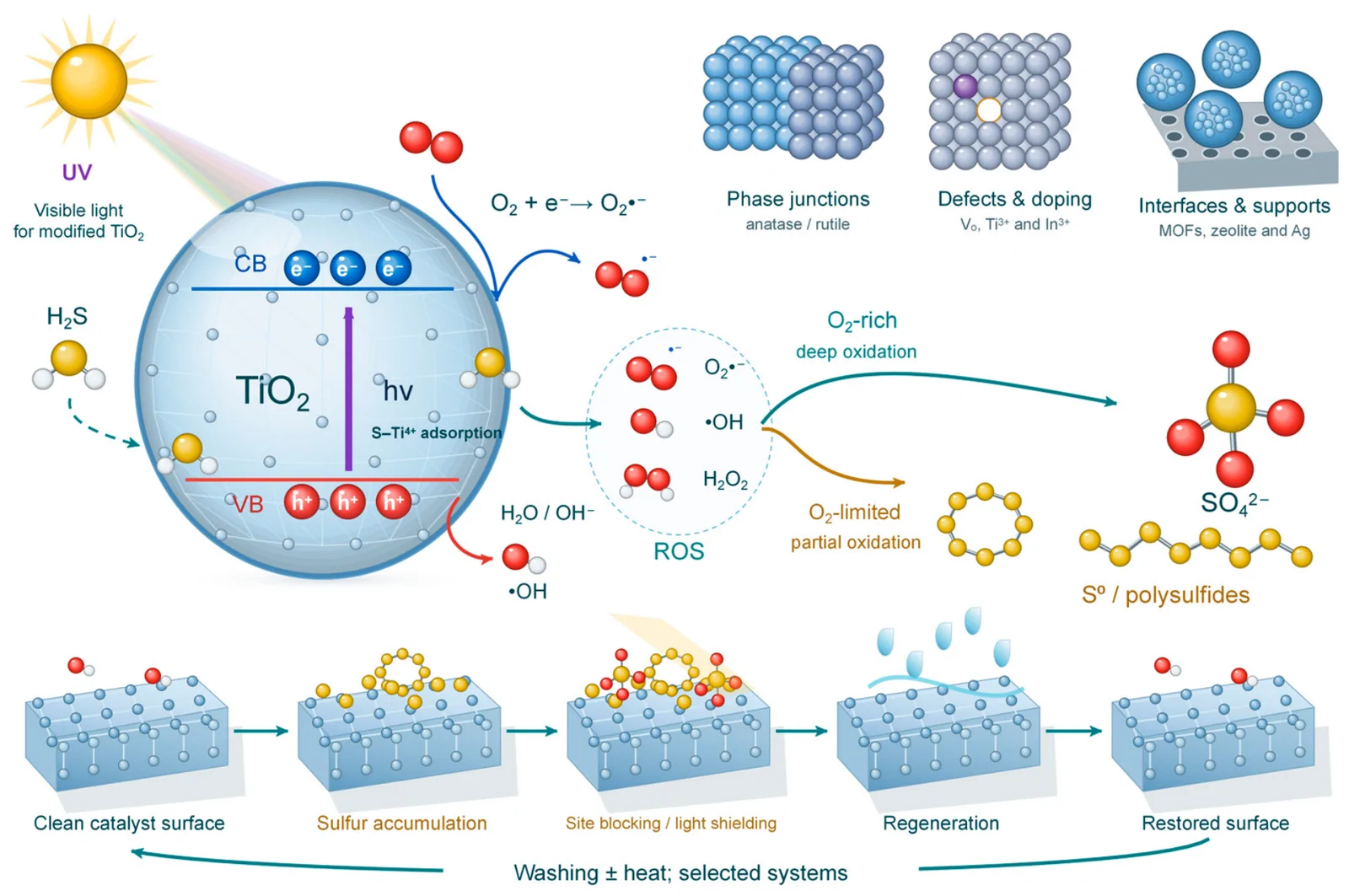
Photocatalytic H_2_S oxidation pathways and deactivation-regeneration of TiO_2_-based photocatalysts.

Photocatalytic H_2_S oxidation over TiO_2_-based catalysts generally proceeds through two competing pathways, as shown in Figure 3: selective partial oxidation to elemental sulfur (S^0^) and deeper oxidation to sulfate (SO_4_^2−^), with product selectivity strongly influenced by surface defects, heterojunction structures, and reaction conditions. However, the accumulation of S^0^ and sulfate species on catalytically active sites can progressively inhibit H_2_S conversion and lead to catalyst deactivation. Various regeneration strategies, including thermal treatment, solvent washing, and chemical etching, have been explored to remove these surface deposits and recover catalytic activity (Figure 3). Nevertheless, maintaining catalyst stability over repeated regeneration cycles while minimizing regeneration energy, chemical consumption, and material loss remains a key challenge for the practical deployment of photocatalytic H_2_S oxidation.

Defect engineering has emerged as an effective strategy for tailoring the photocatalytic properties of TiO_2_. For instance, black TiO_2_ produced via reduction treatment introduces abundant oxygen vacancies and Ti^3+^ defect sites while forming a disordered surface layer. These structural modifications markedly enhance visible-light absorption and facilitate the separation of photogenerated charge carriers [65], providing new avenues for the development of highly efficient TiO_2_-based photocatalysts. To date, multiple optimization strategies, including crystal-phase engineering, defect engineering, elemental doping, and heterostructure construction, have been extensively explored to further improve the photocatalytic performance of TiO_2_-based photocatalysts [66,67].

Canela et al. were the first to systematically demonstrate the crucial role of oxygen in the photocatalytic oxidation of H_2_S over TiO_2_ [68]. Under an air atmosphere (21% O_2_), nearly complete H_2_S conversion (99%) was achieved within 30 min of irradiation using a 30 W black-light lamp. In contrast, under a N_2_ atmosphere, the H_2_S conversion efficiency was only 17%, despite the presence of both TiO_2_ and irradiation. Control experiments confirmed that direct photocatalysis of H_2_S was negligible under the studied conditions. Oxygen not only serves as an electron scavenger to generate superoxide radicals (•O_2_^−^), but also participates in the formation of other ROS, including hydroxyl radicals (•OH) and hydrogen peroxide (H_2_O_2_). This pioneering work established the fundamental role of oxygen in H_2_S photocatalytic oxidation and has been consistently validated, forming a cornerstone for designing H_2_S photocatalytic systems.

Albalawi et al. systematically summarized the product selectivity of TiO_2_-mediated H_2_S photocatalytic oxidation [69]. Under oxygen-rich (aerobic) conditions, H_2_S undergoes complete oxidation via ROS, primarily •OH and •O^2−^, yielding predominantly sulfate species (SO_4_^2−^). In contrast, under oxygen-deficient or oxygen-limited conditions, such as vacuum ultraviolet (VUV)-assisted systems, H_2_S is partially oxidized, producing elemental sulfur (S^0^), polysulfides, and other reduced sulfur intermediates. This distinction explains inconsistencies in product distributions reported in earlier studies and provides a theoretical basis for regulating reaction pathways and product selectivity in H_2_S photocatalytic system [69].

Alonso-Tellez et al. systematically compared the photocatalytic performance of two commercial TiO_2_ photocatalysts, Hombikat UV100 with a high specific surface area and the benchmark P25, for the gas-phase photocatalytic oxidation of H_2_S under UV-A irradiation [70]. Under identical conditions, UV100 maintained complete H_2_S conversion without detectable gaseous SO_2_ emission, whereas P25 exhibited pronounced on-stream deactivation, with the H_2_S conversion declining to approximately 90% after 5 h and 35–40% of sulfur-containing products released as SO_2_. Increasing catalyst loading to 1.77 mg cm^−2^ extended the total sulfur-removal duration of UV100 to 37 h at a gas velocity of 1.4 cm s^−1^, compared with only 6 h for P25. Even at 3.5 cm s^−1^, UV100 remained effective sulfur removal for around 20 h, whereas P25 was active for only 2 h. This performance difference is primarily attributed to the higher specific surface area of UV100, which provides greater storage capacity for sulfate species, delaying active-site blockage and suppressing SO_2_ release [70].

In a subsequent study, Alonso-Tellez et al. further investigated the effect of TiO_2_ P25 coating density on the H_2_S photocatalytic oxidation [71]. Increasing the catalyst loading effectively prolonged the duration of complete H_2_S conversion and delayed the formation of SO_2_. Under conditions of 15 ppm H_2_S and a gas flow rate of 500 mL min^−1^, a low coating density (0.04 mg cm^−2^) achieved only approximately 50% of H_2_S conversion with about 12% SO_2_ selectivity. When the coating density exceeded 0.44 mg cm^−2^, complete H_2_S conversion was attained and SO_2_ selectivity decreased significantly. Notably, non-illuminated inner layers of the TiO_2_ coating contributed by adsorbing SO_2_ and delaying its release, increasing the residence time of both H_2_S and SO_2_ within the coating and mitigating SO_2_ breakthrough [71].

**Figure 4:**
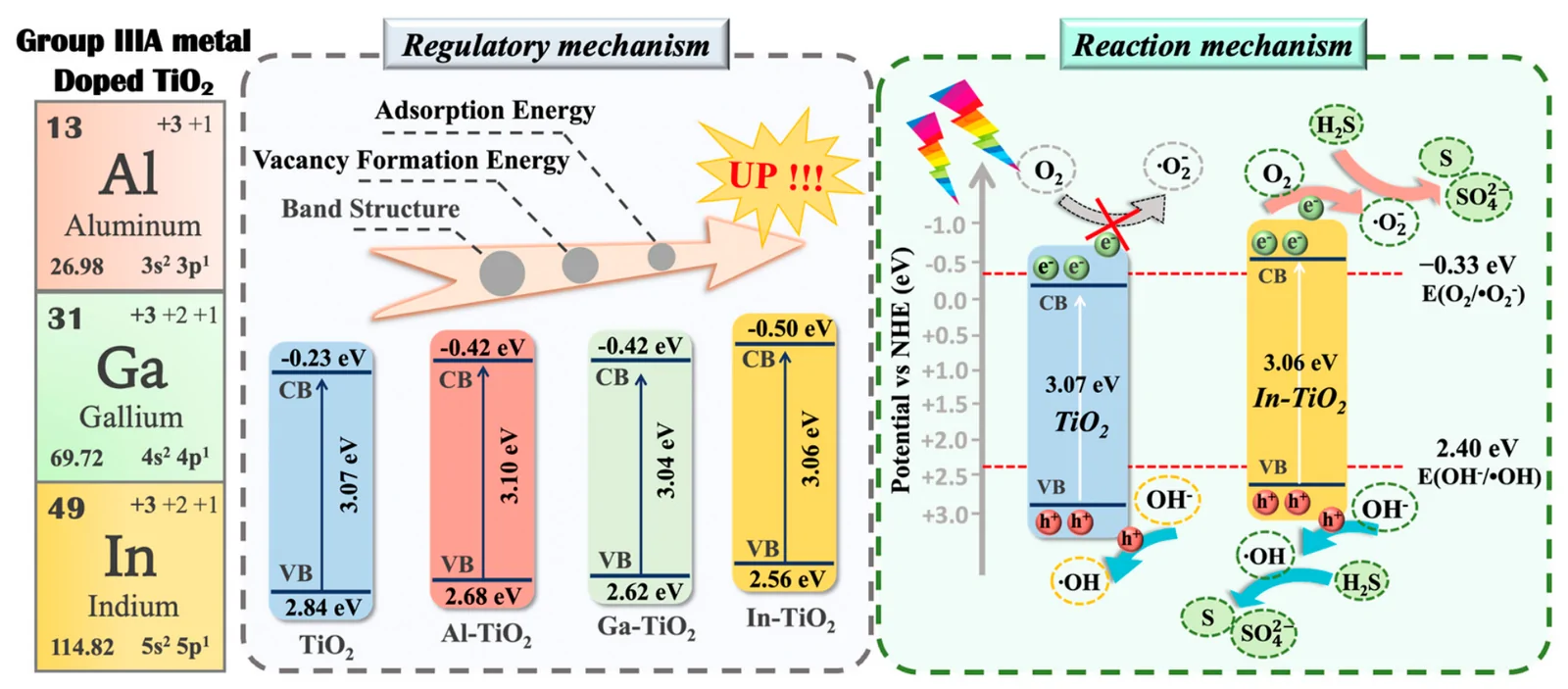
Photocatalytic H_2_S degradation mechanism of Al^3+^-, Ga^3+^-, and In^3+^-doped TiO_2_ photocatalysts [72].

Except for structural optimization, elemental doping has also been widely employed to enhance TiO_2_ photocatalytic activity. Huang et al. synthesized Al^3+^-, Ga^3+^-, and In^3+^-doped TiO_2_ photocatalysts via a Sol–gel method and systematically evaluated their performance for photocatalytic H_2_S oxidation under UV irradiation [72]. All doped samples outperformed pristine TiO_2_ and commercial P25, with 4% In-doped TiO_2_ exhibiting the best performance, maintaining complete H_2_S removal for 120 min, which was three times longer than P25. Combined ultraviolet photoelectron spectroscopy (UPS), valence-band X-ray photoelectron spectroscopy (VB-XPS), and density functional theory (DFT) analyses revealed that the incorporation of group IIIA metal ions introduced shallow acceptor levels above the valence band of TiO_2_, resulting in a shift in the conduction-band edge toward more negative potentials. This facilitated O_2_ reduction by photogenerated electrons to generate superoxide radicals (•O^2−^), thereby enhancing ROS formation and significantly improving H_2_S photocatalytic oxidation (Figure 4) [72].

Alonso-Tellez et al. were the first to incorporate WO_3_ into high-surface-area TiO_2_ (Hombikat UV100) and systematically investigated the effect of WO_3_ loadings ranging from 5 to 20 wt% on the H_2_S photocatalytic oxidation [73]. Under mild conditions (1.77 mg·cm^−2^ catalyst, 200 mL·min^−1^ gas flow), 10 wt% WO_3_/UV100 catalyst exhibited the best performance, increasing the total sulfur-removal duration from 37 h (pristine UV100) to 51 h, a 38% improvement. Although 20 wt% WO_3_/UV100 showed a sulfur-removal duration comparable to that of bare UV100, the SO_2_ release rate decreased markedly from 3.9% h^−1^ to 1.5% h^−1^, indicating that WO_3_ strongly adsorbs SO_2_ but has limited oxidation capability, thereby delaying SO_2_ breakthrough [73].

Brancher et al. immobilized P25 TiO_2_ onto fiberglass filters using an impregnation method, forming a porous nanoparticle film approximately 1.8 μm thick with high surface roughness [74]. The coated filters were integrated into an annular photocatalytic reactor to evaluate the long-term performance of H_2_S photocatalytic oxidation. Under optimized conditions (gas flow rate of 25 L h^−1^, residence time of 121 s, inlet H_2_S concentration of 18 ppm, and relative humidity of 65%), the H_2_S removal efficiency remained above 89% for 16 h and still reached 64% after 28 h of continuous operation without complete catalyst deactivation. These findings highlight the potential of fiberglass-supported TiO_2_ for the continuous treatment of low-concentration H_2_S streams. Moreover, catalyst immobilization eliminates the recovery and handling challenges associated with powdered photocatalysts, offering a practical strategy for reactor scale-up and industrial implementation.

Coronado et al. investigated the influence of support materials on H_2_S photocatalytic oxidation by depositing sol–gel-derived TiO_2_ onto borosilicate glass, polyethylene terephthalate (PET), and cellulose acetate (CA) substrates [75]. Among the tested materials, the uncalcined TiO_2_/CA coating exhibited the highest initial H_2_S conversion (about 65%), followed by uncalcined TiO_2_/PET and TiO_2_/glass coatings, whereas calcined TiO_2_/glass showed the lowest activity (circa 10%). The inferior performance of calcined TiO_2_ was attributed to grain growth during thermal treatment, which reduced the specific surface area and surface hydroxyl density, thereby diminishing photocatalytic activity.

Grzeskowiak et al. developed a three-dimensional photocatalytic system comprising TiO_2_ supported on quartz wool for the UV-A oxidation of high-concentration H_2_S (2000 ppm) [76]. Owing to the high UV-A transmittance of quartz fibers, ultraviolet light penetrated deeply into the composite structure, activating a larger fraction of TiO_2_ particles. Pure TiO_2_ powder reduced the H_2_S concentration by only approximately 10% and became completely deactivated within 200 min. In contrast, the TiO_2_/quartz wool composite maintained stable H_2_S removal for a substantially longer period under dry conditions. Nevertheless, a substantial decline in UV-A transmittance was observed after the reaction, indicating that sulfur deposition not only blocked active sites but also enhanced light-shielding effects, thereby accelerating catalyst deactivation through a positive-feedback mechanism.

Besides deactivation caused by sulfur deposition, catalyst poisoning by H_2_S remains a major challenge in heterogeneous catalysis. Kako et al. investigated the poisoning behavior of H_2_S toward noble-metal catalysts and demonstrated the protective role of TiO_2_ photocatalysis [77]. XPS analyses revealed that, in the absence of illumination, H_2_S readily reacted with Pd surfaces to form metal sulfides, leading to rapid catalyst deactivation. However, under UV irradiation, TiO_2_ photocatalytically oxidized H_2_S into sulfate species, thereby suppressing sulfide formation and preserving catalytic activity.

More recently, Sheng et al. synthesized sea-urchin-like TiO_2_@MIL-101(Cr) double-shell hollow particles using a hard-template approach combined with layer-by-layer self-assembly [78]. The MIL-101(Cr) component provided an exceptionally high specific surface area and strong H_2_S adsorption capacity, while the hollow TiO_2_ shell offered abundant photocatalytically active sites. Under UV irradiation, the TiO_2_@MIL-101(Cr) composite achieved 90.1% H_2_S conversion within 60 min at an inlet H_2_S concentration of 200 ppm, significantly outperforming both hollow TiO_2_ and bulk TiO_2_. In addition, the adsorption–degradation equilibrium time was reduced to 60 min, and the reaction rate nearly doubled relative to hollow TiO_2_. Notably, no significant loss of activity was observed after four consecutive cycles without regeneration. This study represents one of the earliest successful integrations of the strong adsorption capability of MOFs with the photocatalytic oxidation properties of TiO_2_, effectively overcoming the mass-transfer limitations arising from the limited adsorption capacity of conventional TiO_2_ photocatalysts.

Li et al. investigated ozone (O_3_)-assisted photocatalytic oxidation of H_2_S using a TiO_2_ photocatalyst supported on nickel foam and irradiated with a 185 nm ozone-generating UV lamp [79]. The standalone O_3_/UV system (without TiO_2_) achieved only about 30% H_2_S removal after 300 min, whereas the combined O_3_/UV/TiO_2_ system attained a maximum removal efficiency exceeding 97% within 90 min. Moreover, irradiation at 185 nm significantly outperformed 254 and 365 nm wavelengths, owing to its superior O_3_-generation capability. Ozone acts as an efficient electron scavenger, suppressing photogenerated electron–hole recombination while simultaneously decomposing to produce additional hydroxyl radicals (•OH). These results demonstrate that coupling ozone with photocatalysis is an effective strategy for enhancing H_2_S oxidation, particularly under humid conditions.

**Figure 5:**
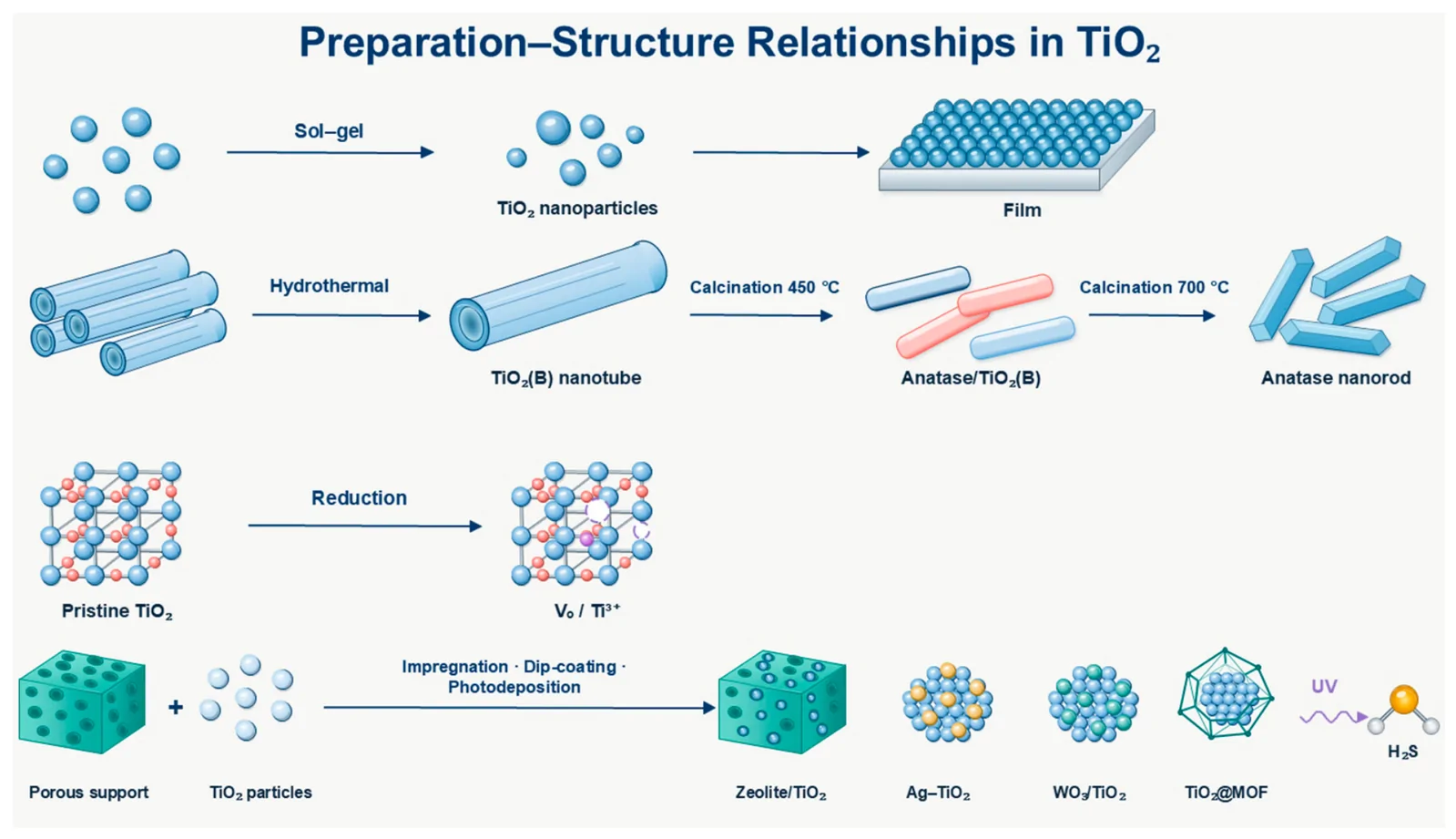
Preparation and structural engineering strategies for TiO_2_-based photocatalysts.

TiO_2_-based photocatalysts can be prepared and structurally engineered through several complementary approaches, as schematically illustrated in Figure 5. Sol–gel processing produces TiO_2_ nanoparticles that can be further assembled into films, whereas hydrothermal synthesis enables the formation of TiO_2_(B) nanotubes, which transform into mixed anatase/TiO_2_(B) phases at 450 °C and anatase nanorods at 700 °C, demonstrating effective control over phase composition and morphology. Reduction treatment introduces oxygen vacancies and Ti^3+^ defects into pristine TiO_2_, providing an additional means of tuning its electronic structure. TiO_2_ can also be immobilized on porous supports or integrated with other functional components through impregnation, dip-coating, or photodeposition, yielding architectures such as zeolite/TiO_2_, Ag-TiO_2_, WO_3_/TiO_2_, and TiO_2_@MOF. Overall, these synthetic strategies enable systematic engineering of TiO_2_ morphology, crystal phase, surface defects, and heterointerfaces, all of which are important for optimizing photocatalytic performance in applications such as UV-driven H_2_S conversion.

Kato et al. prepared Ag nanoparticle-deposited TiO_2_ photocatalytic filter materials (Ag-PCFs) via a photodeposition method and evaluated their performance for H_2_S photocatalytic oxidation under UV irradiation [80]. Ag deposition markedly improved photocatalytic activity. Specifically, Ag-PCF(1.0) achieved an H_2_S removal efficiency of approximately 80%, compared with only 11% for the pristine photocatalytic filter material, corresponding to an approximately sevenfold increase in the degradation rate. XPS and TEM analyses confirmed the presence of uniformly dispersed metallic Ag and Ag_2_O nanoparticles on the TiO_2_ surface. No significant loss of activity was observed after 12 h of continuous operation, and XPS results indicated that the oxidation state of Ag remained unchanged throughout the reaction. Furthermore, Ag-PCF(1.0) exhibited appreciable H_2_S adsorption under dark conditions, whereas samples with lower Ag loadings showed negligible adsorption. These results suggest that Ag serves as an effective cocatalyst by enhancing H_2_S adsorption near the TiO_2_ surface and facilitating photogenerated charge separation, thereby promoting sulfate formation during photocatalytic oxidation.

To improve catalyst immobilization and facilitate practical reactor applications, Portela et al. deposited TiO_2_ sol onto glass Raschig rings via dip-coating and controlled the film thickness by varying the number of coating cycles [81]. The resulting materials were evaluated for H_2_S photocatalytic oxidation. However, excessively thick coatings compromised mechanical stability and increased preparation time, highlighting the need to balance catalytic performance with practical considerations.

In a subsequent study, Portela et al. compared the H_2_S photocatalytic oxidation performance for TiO_2_ immobilized on three support materials of glass Raschig rings, polyethylene terephthalate (PET), and cellulose acetate (CA) monolithic honeycomb structures [82]. Among these supports, TiO_2_-coated PET exhibited the highest initial photocatalytic activity of H_2_S and the best long-term stability, while also demonstrating excellent regenerability through repeated water-washing cycles. Although TiO_2_-coated CA initially showed relatively high activity, weak adhesion between the TiO_2_ layer and the polymer substrate, together with oxidative degradation of the CA support by photocatalytically generated reactive oxygen species, led to substantial performance deterioration after repeated regeneration. Glass Raschig rings also exhibited high photocatalytic activity; however, direct comparison with polymeric monoliths was complicated by differences in reactor geometry and available coating surface area [82].

Uesugi et al. synthesized TiO_2_ nanotubes via a two-step hydrothermal method and tailored their crystal phases through calcination at different temperatures [83]. X-ray diffraction (XRD) and TEM analyses revealed that calcination at 350 °C yielded pure TiO_2_(B) nanotubes, while treatment at 450 °C produced an anatase/TiO_2_(B) mixed-phase structure. Further increasing the calcination temperature to 700 °C transformed the nanotubes into anatase-dominated nanorods. The corresponding specific surface areas were 303, 277, and 57 m^2^ g^−1^, respectively. Under UV irradiation (254 nm) and an H_2_S concentration of 10 ppm in air, the sample calcined at 450 °C exhibited the highest photocatalytic activity. This superior performance was attributed to its relatively high specific surface area, which provided abundant adsorption and reaction sites, as well as the formation of a type-II heterojunction between anatase and TiO_2_(B). The heterojunction promoted electron transfer from anatase to TiO_2_(B) and hole migration in the opposite direction, thereby effectively suppressing charge-carrier recombination [83].

Hao et al. evaluated the regeneration performance of zeolite/TiO_2_ composite photocatalysts using a strategy that combined alkaline washing with NaOH solution (pH = 10) and subsequent calcination at 400 °C for 2 h [84]. After five regeneration cycles, the H_2_S removal efficiency of Reactor C (containing zeolite/TiO_2_) decreased only slightly from 97% to 91%, significantly outperforming Reactor A (containing zeolite) and Reactor D (containing zeolite/TiO_2_ and TiO_2_ film). The alkaline treatment imparted a negative surface charge to TiO_2_, facilitating sulfate desorption, while calcination improved TiO_2_ dispersion on the zeolite support and restored the specific surface area and pore volume. These results demonstrate that the proposed regeneration method is a simple and effective approach for sustaining the long-term performance of zeolite/TiO_2_ photocatalysts in biogas purification applications [84].

Similarly, Liu et al. prepared a composite photocatalyst by coating anatase TiO_2_ onto natural zeolite and evaluated its performance for H_2_S oxidation in simulated biogas streams [85]. Compared with pure zeolite and pristine TiO_2_, the composite catalyst exhibited a superior H_2_S removal capacity of 0.10 mmol g^−1^ under dry conditions, together with the lowest SO_2_ emission. Under humid conditions, the removal capacity further increased to 0.13 mmol g^−1^. The high specific surface area and alkaline nature of zeolite enhanced H_2_S adsorption and enrichment while promoting the capture and subsequent oxidation of SO_2_ intermediates into sulfate species, thereby mitigating secondary pollution. Although CO_2_ strongly competed with H_2_S for adsorption sites on zeolite, it exerted minimal influence on the TiO_2_ photocatalytic oxidation pathway. Consequently, the synergistic combination of zeolite adsorption and TiO_2_ photocatalysis enabled efficient and selective H_2_S removal even in CO_2_-rich biogas environments [85].

To elucidate the reaction mechanism of H_2_S photocatalytic oxidation, Kataoka et al. employed in situ transmission Fourier-transform infrared spectroscopy (FT-IR) to monitor the evolution of surface species on ultrathin TiO_2_ films (<0.3 μm) in real time [86]. In addition to the gradual accumulation of sulfate species, two weak absorption bands at 1093 and 996 cm^−1^ were detected. Their intensities reached a maximum after approximately 60 min and subsequently declined. Comparison with reference spectra in the literature assigned these bands to SO_2_^−^ (sulfite radical anion) rather than SO_3_^−^, suggesting that SO_2_^−^ is a key intermediate in the eight-electron oxidation pathway from H_2_S (S^2−^) to sulfate (S^6+^). In contrast, conventional diffuse reflectance infrared Fourier-transform spectroscopy (DRIFTS) detected only sulfate accumulation and failed to capture this transient intermediate, highlighting the unique capability of in situ FT-IR spectroscopy for mechanistic investigations [86].

Junkaew et al. compared two competing reaction pathways for H_2_S conversion on TiO_2_ surfaces: pathways leading to H_2_O formation (Path IA/IIA) and pathways producing H_2_ (Path IB/IIB) [87]. DFT calculations revealed that the rate-determining steps associated with H_2_O formation possessed relatively low activation barriers (11–13 kcal·mol^−1^) and were thermodynamically exothermic. In contrast, the pathways leading to H_2_ production required substantially higher activation energies (67–88 kcal·mol^−1^) and were endothermic. Consequently, TiO_2_(001) exhibited a strong kinetic and thermodynamic preference for H_2_O formation. These theoretical predictions are consistent with experimental observations, in which sulfate and water are the predominant products of H_2_S photocatalytic oxidation over TiO_2_, whereas H_2_ generation is negligible [87].

Liu et al. synthesized Mn-, Cu-, Ni-, and Co-doped TiO_2_ photocatalysts and systematically evaluated their H_2_S degradation performance under conventional UV photocatalytic oxidation (UV-PCO, 254 nm) and vacuum ultraviolet photocatalytic oxidation (VUV-PCO, 185 and 254 nm) [88]. Under UV-PCO conditions, H_2_S removal efficiencies were extremely low, reaching only 8.1% for pristine TiO_2_ and less than 5% for all doped samples. In contrast, VUV-PCO dramatically enhanced H_2_S removal, achieving efficiencies of 71.0–89.9%. Among the catalysts, 1% Mn-TiO_2_ exhibited the highest activity, attaining an H_2_S removal efficiency of 89.9% without detectable residual ozone in the outlet stream. Quantitative analysis showed that the overall VUV-PCO performance arose from direct VUV photolysis (68.1%), photocatalytic oxidation (3.0%), and ozone catalytic oxidation (18.7%). The superior performance of Mn-TiO_2_ was attributed to the presence of MnO_2_ species, which efficiently decomposed O_3_ into highly reactive O(^1^D) and •OH radicals, thereby promoting H_2_S oxidation [88].

Lied et al. compared the H_2_S photocatalytic oxidation performance of two commercial TiO_2_-based photocatalysts, Ag-containing Fotosan^®^ and pure anatase InterBrasil^®^ FA-101, under both UV and solar irradiation [89]. When coated onto fiber-cement roofing tiles, Fotosan^®^ achieved H_2_S removal efficiencies of 77.4% under UV irradiation (gas flow rate of 2 L·min^−1^, residence time of 115 s) and 74.9% under solar irradiation. In contrast, InterBrasil^®^ achieved only 40.9% removal under UV light, which further decreased to 19.8% under solar irradiation. XRD analysis confirmed the presence of Ag nanoparticles in Fotosan^®^, which enhanced charge separation by trapping photogenerated electrons while simultaneously reducing UV-Vis reflectance and improving light harvesting [89].

In a subsequent study, Lied et al. investigated a commercial rutile-based TiO_2_ coating, which differs from the anatase and P25 photocatalysts commonly used in H_2_S photocatalytic oxidation [90]. Under optimized conditions, the catalyst achieved an H_2_S removal efficiency of up to 94%. However, increasing the gas flow rate to 14 L·min^−1^ caused the removal efficiency to decline sharply to 33%, because the shorter gas–solid contact time limited mass transfer and surface reaction kinetics. By contrast, the inlet H_2_S concentration had a relatively minor effect on performance, suggesting that the photocatalytic oxidation process followed pseudo-first-order kinetics under the investigated conditions [90].

Lafjah et al. synthesized a series of anatase TiO_2_ nanorods via alkaline hydrolysis of commercial P25, followed by calcination at varying temperatures [91]. As the calcination temperature increased from 350 to 700 °C, the BET specific surface area decreased from 376 to 47 m^2^·g^−1^, whereas the crystallite size increased from 8 to 23 nm. All samples retained a pure anatase phase. Among them, the nanorods calcined at 380 °C exhibited the highest photocatalytic performance, maintaining 64% H_2_S conversion and 51% sulfur removal after 300 min. By comparison, commercial P25 achieved only 25% H_2_S conversion and 20% sulfur removal under the same conditions. Although the sample calcined at 350 °C had a higher specific surface area, its lower crystallinity limited H_2_S conversion to approximately 35%. Samples calcined above 500 °C experienced accelerated deactivation due to substantial surface-area loss. These results indicate that, in sulfate-sensitive photocatalytic systems, high specific surface area is more critical than crystallinity for sustaining long-term activity [91].

Monteiro et al. prepared nitrogen-doped titanate nanotubes (N1TiNT-400) using urea as the nitrogen source and evaluated their photocatalytic H_2_S oxidation under UVA and solar irradiation [92]. Under UVA illumination, the catalyst achieved 98% H_2_S conversion within 23 min with low SO_2_ selectivity. Under solar irradiation, conversion decreased to 78%, accompanied by a rise in SO_2_ selectivity to around 40%. This was attributed to the lower UVA fraction in solar light, which limited the generation of photogenerated electron–hole pairs and reactive oxygen species (•OH and •O^2−^), leading to incomplete oxidation of intermediates such as SO_2_.

Sun et al. applied Hg-modified TiO_2_ photocatalysts in a portable air purification system and investigated the effect of Hg loading on H_2_S removal [93]. The optimal catalyst, containing 2 wt% Hg, achieved 99.3% H_2_S removal within 360 s, corresponding to a clean air delivery rate of 17.9 L min^−1^, a reaction rate of 1.75 mmol·h^−1^·g^−1^, and a quantum yield of 2.04 × 10^−1^ molecules photon^−1^, all significantly higher than pristine TiO_2_. Electrochemical impedance spectroscopy (EIS) and photoluminescence (PL) analyses confirmed that Hg incorporation reduced charge-transfer resistance and suppressed electron–hole recombination, enhancing photocatalytic activity. These findings demonstrate the effectiveness of Hg as a cocatalyst for H_2_S oxidation over TiO_2_.

Portela et al. fabricated TiO_2_/Cr-MCM-41 composites by incorporating Cr into mesoporous MCM-41 and loading 20 wt% TiO_2_ [94]. UV–Vis diffuse reflectance spectroscopy (UV–Vis DRS) showed strong absorption in the visible region (400–600 nm), with an apparent band gap of approximately 2.0 eV. Under visible-light irradiation, TiO_2_/Cr-MCM-41 achieved H_2_S conversion efficiencies two- to threefold higher than under UVA, with negligible SO_2_ formation. In contrast, TiO_2_/MCM-41 and P25-SiO_2_ without Cr exhibited pronounced SO_2_ generation. These results indicate that Cr incorporation not only extends the photoresponse of TiO_2_ into the visible range but also favors complete H_2_S oxidation to sulfate, reducing secondary pollution. Rapid catalyst deactivation, however, occurred due to sulfate accumulation and gradual reduction in Cr^6+^ during operation [94].

**Figure 6:**
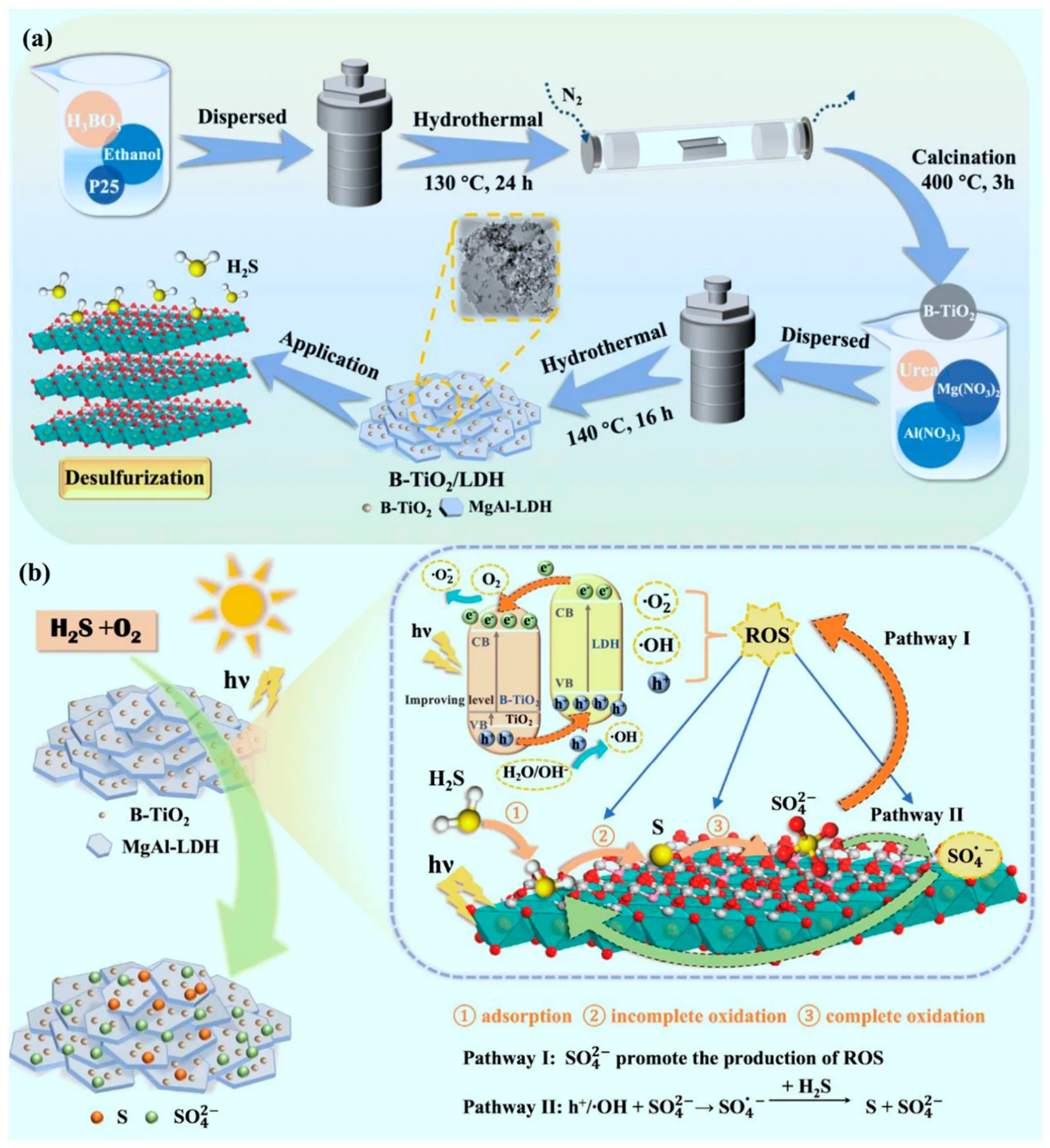
(**a**) Schematic illustration of the synthesis route and heterojunction construction of B-TiO_2_/LDH nanocomposites for visible-light-driven H_2_S photocatalytic oxidation. (**b**) Schematic illustration of the proposed mechanisms for photocatalytic removing H_2_S on the B-TiO_2_/LDH nanocomposite [95].

Huang et al. synthesized a visible-light-responsive heterojunction photocatalyst, boron-doped TiO_2_ coupled with MgAl-layered double hydroxides (B-TiO_2_/LDH), via a hydrothermal method (Figure 6a) [95]. Under visible light irradiation (λ > 420 nm), the 50% B-TiO_2_/LDH composite achieved complete H_2_S removal within 120 min, outperforming P25, B-TiO_2_, and pristine LDH. UV–Vis DRS analysis showed that boron doping shifted the absorption edge of TiO_2_ toward 750 nm, while coupling with LDH further enhanced visible-light absorption. Photocurrent measurements revealed that the heterojunction generated substantially higher photocurrent densities than either component alone, confirming efficient spatial separation of photogenerated charge carriers. During a 40-h stability test, the catalyst maintained 100% H_2_S removal for the first 20 h and >80% thereafter, demonstrating excellent long-term stability under visible-light irradiation. A photothermal synergistic effect in the B-TiO_2_/LDH heterostructure was proposed to enhance the catalytic oxidation of H_2_S. Further investigations revealed that surface-accumulated sulfate species could serve as additional active sites for H_2_S adsorption and subsequent oxidation, thereby promoting the overall catalytic oxidation process (Figure 6b) [95].

### 2.2. Composite Photocatalysts

Although TiO_2_-based photocatalysts have been extensively studied for H_2_S oxidation and have provided fundamental insights into photocatalytic mechanisms, their practical desulfurization performance is constrained by intrinsic limitations, including a wide band gap (circa 3.2 eV), limited visible-light absorption, and rapid recombination of photogenerated charge carriers. To address these challenges, considerable efforts have focused on designing composite photocatalysts by integrating two or more materials with complementary band structures and functional properties. Through interfacial coupling and synergistic interactions, composite systems can extend the photo-response range, enhance charge-carrier separation, and generate abundant surface-active sites, thereby substantially improving photocatalytic H_2_S oxidation performance [96].

**Figure 7:**
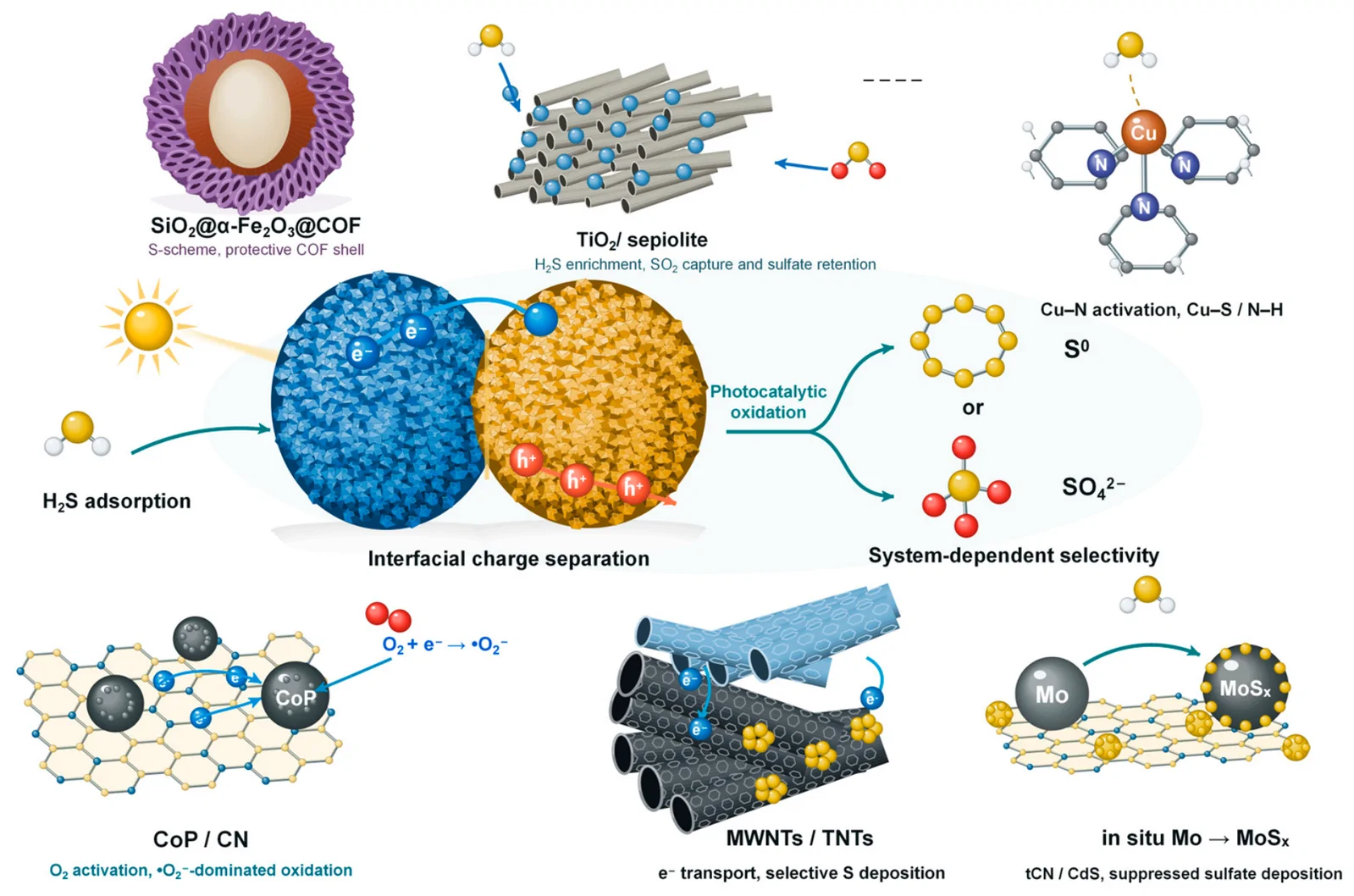
Representative composite photocatalysts and their functional roles in photocatalytic H_2_S removal.

Various representative composite photocatalysts have been developed to enhance photocatalytic H_2_S removal by promoting interfacial charge separation and optimizing reaction pathways, as schematically illustrated in Figure 7. These systems employ distinct functional components to regulate H_2_S adsorption, charge transfer, reactive oxygen species generation, and product selectivity. For example, SiO_2_@α-Fe_2_O_3_@COF utilizes an S-scheme charge-transfer mechanism together with a protective COF shell, whereas TiO_2_/sepiolite promotes H_2_S enrichment while facilitating SO_2_ capture and sulfate retention. Other systems, such as CoP/CN, enhance O_2_ activation to generate •O_2_^−^, thereby favoring H_2_S oxidation, while MWNTs/TNTs facilitate interfacial electron transport and promote selective deposition of elemental sulfur. In addition, specific metal sites, such as Cu-N coordination environments or in situ-generated MoS_x_ species, can regulate the reaction pathway and product selectivity, favoring either elemental sulfur (S^0^) or sulfate (SO_4_^2−^) formation while mitigating excessive sulfate accumulation.

#### 2.2.1. Heterojunction Photocatalysts

**Figure 8:**
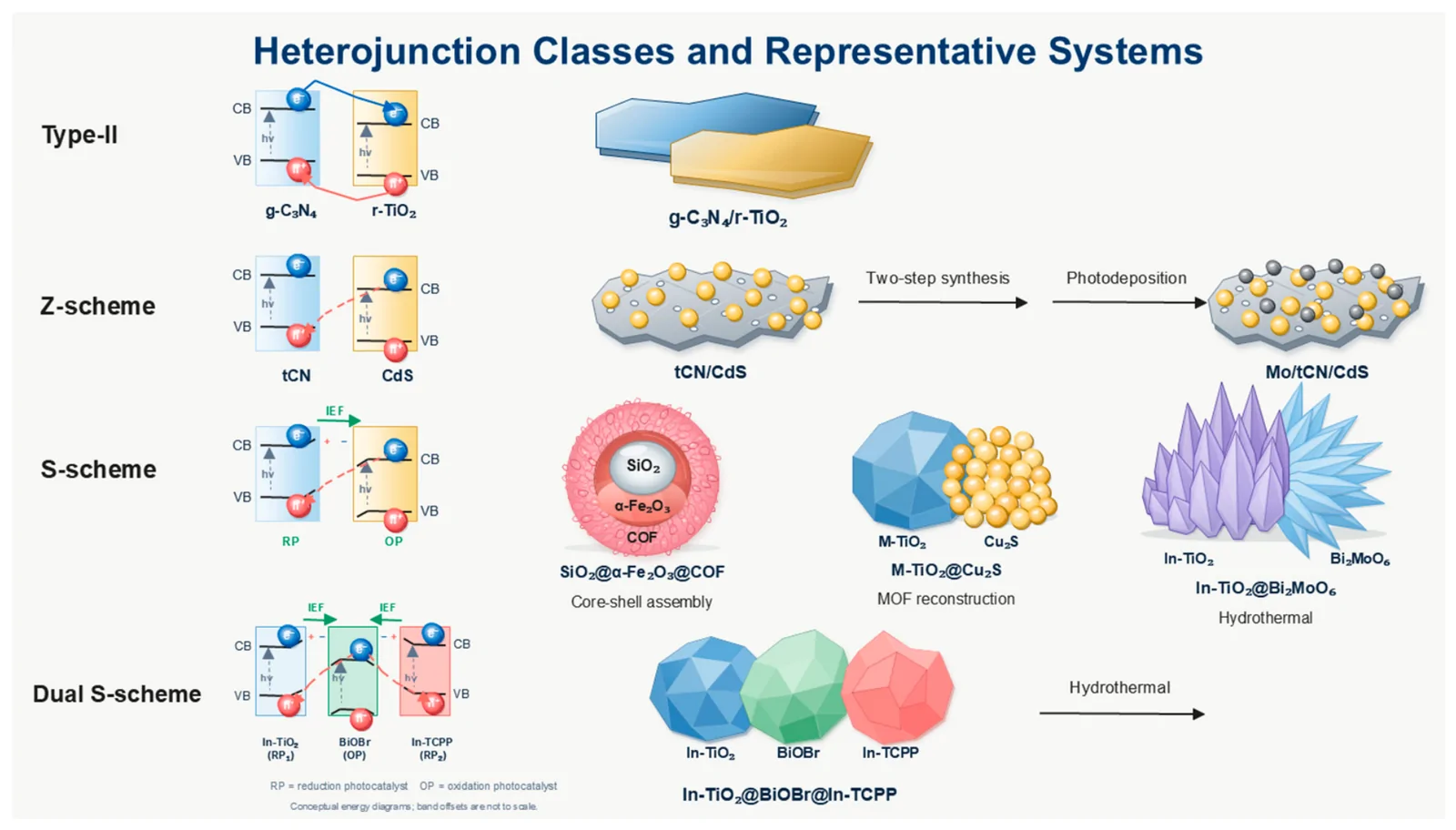
Heterojunction configurations and representative photocatalysts for H_2_S removal.

Heterojunction photocatalysts have attracted considerable attention because coupling two semiconductors facilitates the spatial separation of photogenerated electrons and holes, suppresses charge-carrier recombination, and enhances photocatalytic performance through efficient interfacial charge transfer [97]. Moreover, incorporating narrow-band-gap semiconductors extends the photoresponse into the visible-light region, thereby improving solar-energy utilization [98]. Based on their band alignment and interfacial charge-transfer mechanisms, heterojunction photocatalysts are generally classified into Type-II, Z-scheme, and S-scheme systems. In conventional Type-II heterojunctions, photogenerated electrons migrate from the conduction band (CB) of one semiconductor to the lower-lying CB of the other, while holes transfer in the opposite direction, as shown in Figure 8. Although this configuration effectively promotes charge separation, it inevitably weakens the redox capability because the remaining electrons and holes possess less negative and less positive potentials, respectively. In contrast, Z-scheme heterojunctions preserve strong oxidation and reduction abilities by allowing the recombination of low-energy electrons and weakly oxidative holes, while retaining highly reductive electrons and highly oxidative holes. Nevertheless, conventional Z-scheme systems often require electron mediators or rely on indirect charge-transfer pathways, which may increase system complexity and reduce charge-transfer efficiency. More recently, S-scheme heterojunctions have emerged as an advanced charge-transfer model. In these systems, band bending, an interfacial built-in electric field, and Coulomb interactions collectively drive the selective recombination of low-energy charge carriers while preserving highly energetic electrons and holes with strong redox potentials. Consequently, S-scheme heterojunctions are particularly promising for photocatalytic H_2_S oxidation, where efficient generation of ROS and strong oxidative capability are crucial for promoting H_2_S activation and the subsequent conversion of sulfur-containing intermediates into stable oxidation products [99,100].

Pan et al. constructed a SiO_2_@α-Fe_2_O_3_@COF sandwich-structured core–shell S-scheme heterojunction photocatalyst through interfacial engineering. SiO_2_ nanospheres served as the core, providing structural stability and high surface area; α-Fe_2_O_3_ formed the intermediate layer; and a porous covalent organic framework (TAPB-DMTP-COF, BET surface area of 602 m^2^ g^−1^) constituted the outer shell. Under visible-light irradiation, this photocatalyst achieved 95.2% H_2_S conversion at 20 ppm, outperforming SiO_2_@COF (75.2%) and SiO_2_@α-Fe_2_O_3_. More importantly, the catalyst retained above 92% removal efficiency after five cycles. In contrast, the control SiO_2_@COF@α-Fe_2_O_3_, with α-Fe_2_O_3_ as the outer layer, rapidly deactivated due to sulfur accumulation. These results highlight the COF shell’s dual role in charge transfer and protection of the α-Fe_2_O_3_ active phase, enhancing long-term stability [101].

In a photoelectrochemical H_2_S splitting system, Wang et al. designed a dual-interface heterojunction photoanode consisting of one-dimensional TiO_2_ nanorod arrays, ZnIn_2_S_4_ nanoflowers, and Cu-doped CdIn_2_S_4_ nanosheets. TiO_2_ nanorods functioned as electron-transport pathways, while a Type-II heterojunction formed between TiO_2_ and ZnIn_2_S_4_ promoted electron transfer to TiO_2_ and hole migration in the opposite direction. Simultaneously, electrons in the conductive band (CB) of CdIn_2_S_4_ recombined with holes in the valence band (VB) of ZnIn_2_S_4_, forming an S-scheme heterojunction and retaining highly oxidative holes on the CdIn_2_S_4_ surface [102]. Although this study focused on photocatalytic H_2_S splitting for H_2_ production rather than aerobic oxidation, the dual-interface heterojunction strategy provides valuable insights for the design of highly efficient H_2_S oxidation photocatalysts.

Wang et al. employed an amino-functionalized Ti-based MOF (NH_2_-MIL-125) as a precursor to construct an M-TiO_2_@Cu_2_S S-scheme heterojunction (TCM2) via a thioacetamide-assisted solvothermal process [103]. The amino groups anchored Cu^2+^ ions, directing the homogeneous nucleation of Cu_2_S nanoparticles during thermal transformation, while simultaneously forming mesoporous TiO_2_ (M-TiO_2_) and partially retaining the porous structure of MOF. The resulting material exhibited a high specific surface area of 527.8 m^2^ g^−1^ and abundant channels for H_2_S adsorption and diffusion. High-resolution TEM (HRTEM) confirmed intimate heterointerfaces between M-TiO_2_ and Cu_2_S. DFT calculations indicated that the work function of M-TiO_2_ (4.969 eV) exceeded that of Cu_2_S (3.203 eV), driving spontaneous electron transfer from Cu_2_S to M-TiO_2_ and establishing an internal electric field at the interface. Under visible-light irradiation, TCM2 achieved over 97% H_2_S removal during a 6 h continuous operation, demonstrating excellent photocatalytic activity and stability [103].

A fundamental understanding of heterojunction photocatalytic mechanisms requires elucidating band alignment and charge-transfer pathways at the atomic scale. Wei et al. systematically investigated the electronic structure and interfacial charge-transfer behavior of a g-C_3_N_4_/r-TiO_2_ heterojunction using DFT calculations. The work functions of g-C_3_N_4_ (001) and r-TiO_2_(110) were determined to be 4.90 and 7.44 eV, respectively. Upon contact, electrons spontaneously transferred from g-C_3_N_4_ to r-TiO_2_, generating an internal electric field directed from g-C_3_N_4_ to r-TiO_2_ and resulting in an interfacial charge transfer of approximately 0.14 e. Under light irradiation, the heterojunction exhibits a staggered Type-II charge-transfer mechanism: photogenerated electrons in the CB of r-TiO_2_ recombine with photogenerated holes in the VB of g- C_3_N_4_. As a result, highly reductive electrons are retained in the CB of g-C_3_N_4_ (−1.71 V), while strongly oxidative holes remain in the VB of r-TiO_2_ (+3.22 V), promoting efficient photocatalytic redox reactions [104].

**Figure 9:**
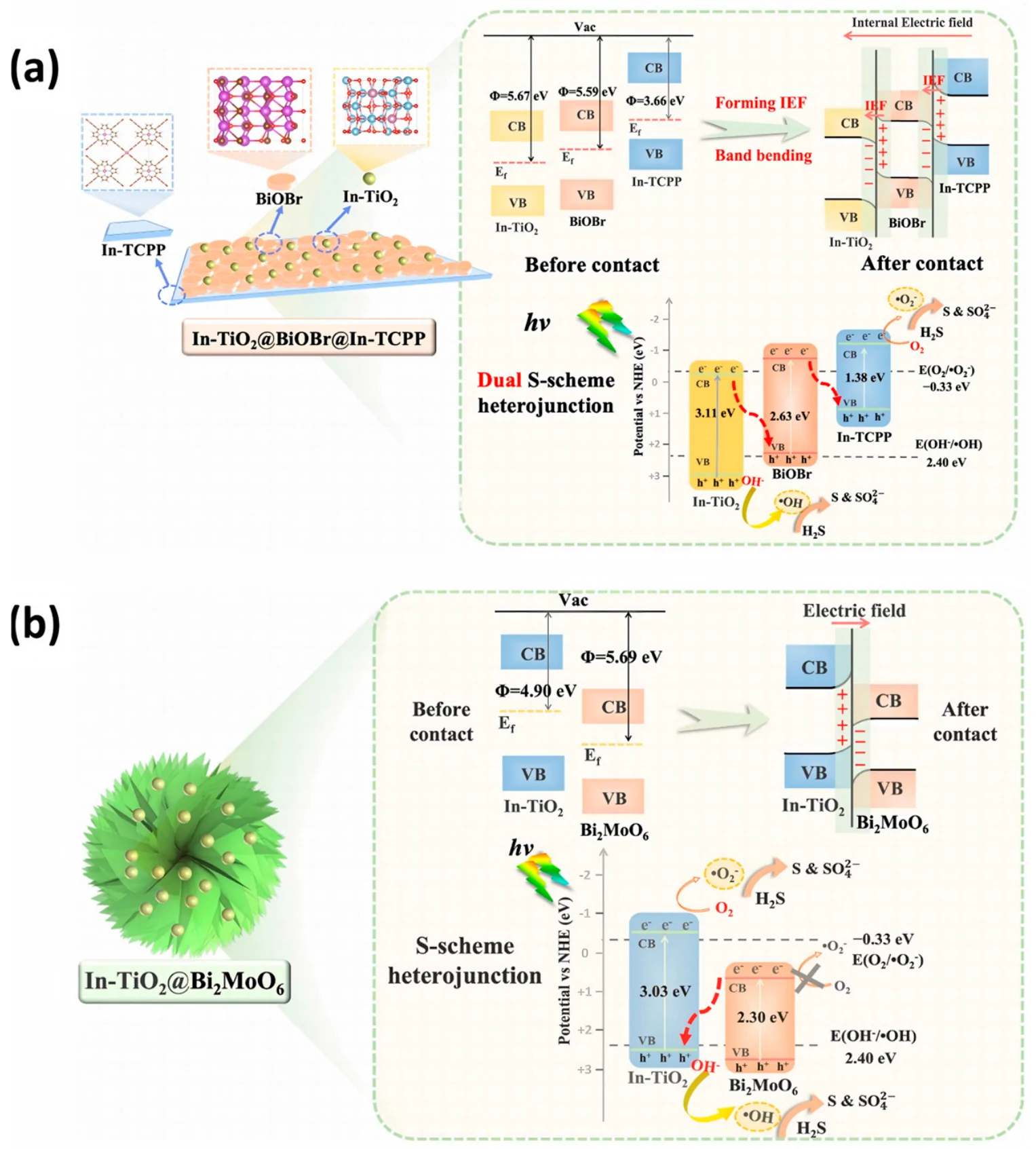
(**a**) Proposed photocatalytic oxidation pathway of H_2_S over the IBP-3 dual S-scheme heterojunction photocatalyst under visible-light irradiation [105]. The “+” and “−” symbols represent positively and negatively charged regions, respectively, arising from interfacial charge redistribution upon contact. (**b**) Schematic illustration of the charge-separation and photocatalytic oxidation mechanism of In-TiO_2_@Bi_2_MoO_6_ S-scheme heterojunction photocatalyst [106].

Huang et al. synthesized a novel ternary dual S-scheme heterojunction photocatalyst, In-TiO_2_@BiOBr@In-TCPP (IBP), via a hydrothermal method for pronounced visible-light-driven H_2_S photocatalytic oxidation. In this architecture, In^3+^-doped anatase TiO_2_ (3.11 eV) served as the primary active component, providing sites for H_2_S dissociation, while BiOBr (2.63 eV) acted as an electron-transfer mediator. The porphyrin-based MOF In-TCPP (1.38 eV) significantly broadened the photoresponse range to approximately 1200 nm (Figure 9a). Benefiting from the synergistic effects of the dual S-scheme heterojunction, the optimized IBP-3 photocatalyst achieved complete removal of 100 ppm H_2_S within 120 min under visible-light irradiation, markedly outperforming the individual components; under identical conditions, In-TiO_2_ achieved only 15.8% conversion. Moreover, H_2_S removal efficiencies exceeding 86% were maintained across a broad concentration range (50–250 ppm) and gas hourly space velocities (GHSV) of 3500–15,500 h^−1^. During an 8 h continuous operation test, the catalyst retained more than 70% of its initial activity and exhibited excellent structural stability after the reaction. These results demonstrate that the dual S-scheme architecture effectively enhances visible-light harvesting, charge-carrier separation, and photocatalytic H_2_S oxidation performance [105].

Wang et al. synthesized flower-like In-TiO_2_@Bi_2_MoO_6_ S-scheme heterojunction photocatalysts via a hydrothermal method for visible-light-driven H_2_S oxidation (Figure 9b). The integration of layered Bi_2_MoO_6_ (2.30 eV) with In^3+^-doped anatase TiO_2_ (3.03 eV) induced a pronounced redshift in the optical absorption edge. The composite also exhibited enhanced photocurrent responses and stronger PL quenching compared with the individual components, indicating improved charge-carrier separation. The optimized In-TiO_2_-0.4@Bi_2_MoO_6_ catalyst achieved complete removal of 100 ppm H_2_S within 120 min under visible-light irradiation (λ > 420 nm), whereas pristine In-TiO_2_ and Bi_2_MoO_6_ removed only 15.8% and 44.8%, respectively. Even at an elevated H_2_S concentration of 250 ppm, the removal efficiency exceeded 90%, and a high GHSV of 22,500 h^−1^ still yielded 73.2% removal. During an 8 h continuous operation test, complete H_2_S removal was maintained for the first 6 h, and the catalyst retained more than 65% activity after 8 h. These results demonstrate that binary S-scheme heterojunctions effectively enhance visible-light activity, charge separation, and resistance to sulfur-induced deactivation, while the straightforward synthesis procedure underscores their potential for practical H_2_S abatement applications [106].

#### 2.2.2. Other Composite Photocatalysts

Katayama and Nagata prepared a g-C_3_N_4_/CdS composite photocatalyst via a two-step synthesis route for visible-light-driven decomposition of low-concentration H_2_S [107]. Porous g-C_3_N_4_ (tCN), synthesized from thiourea, served as the support for uniformly dispersed CdS nanoparticles, forming a Z-scheme heterojunction. Under visible-light irradiation (λ > 400 nm), the tCN/CdS composite reduced 10 ppm H_2_S to 0.1 ppm within 30 min, significantly outperforming pristine g-C_3_N_4_, pure CdS, and commercial P25 under UV light irradiation. The enhanced photocatalytic activity is attributed to the narrow band gap of CdS (about 2.4 eV), enabling efficient visible-light absorption, and the Z-scheme charge-transfer pathway between g-C_3_N_4_ and CdS. This heterojunction effectively promotes the spatial separation of photogenerated charge carriers, suppresses electron–hole recombination, and thereby enhances photocatalytic activity [107].

Building on this system, Katayama and Nagata further introduced a Mo cocatalyst onto the g-C_3_N_4_/CdS composite via photodeposition, producing a ternary photocatalyst denoted as TCM for the visible-light-driven oxidation of low-concentration gaseous H_2_S [108]. Compared with the Mo-free tCN/CdS catalyst, TCM reduced H_2_S concentrations below the detection limit within 30 min and maintained high removal efficiency over 180 min of continuous operation. In contrast, the activity of the unmodified catalyst gradually declined due to sulfate accumulation on the catalyst surface. XPS analysis revealed a pronounced S^6+^ sulfate signal on the spent tCN/CdS catalyst, whereas only a negligible sulfate peak was detected on TCM. These findings indicate that Mo preferentially reacts with H_2_S to form MoS_x_ species, thereby mitigating sulfate deposition on the CdS surface. Moreover, the in situ generated MoSx species exhibit intrinsic photocatalytic activity and further promote H_2_S decomposition, resulting in a synergistic effect that enhances both the activity and stability of the composite photocatalyst [108].

Rasmussen et al. combined natural sepiolite (SiMgOx) with TiO_2_ to fabricate plate-shaped composite photocatalysts via an extrusion process for the integrated adsorption and photocatalytic oxidation of H_2_S. Although pure P25 TiO_2_ exhibited high initial activity toward H_2_S oxidation, gaseous SO_2_ began to emerge after approximately 1 h of operation, and its concentration progressively increased until nearly all sulfur was converted to SO_2_. In contrast, the TiO_2_-SiMgOx composite completely suppressed SO_2_ emissions for at least 12 h while maintaining an H_2_S removal efficiency comparable to that of P25. The high specific surface area and alkaline surface characteristics of sepiolite not only enhanced H_2_S adsorption but also facilitated the capture of SO_2_ intermediates generated during photocatalytic oxidation. These intermediates were subsequently oxidized to sulfate species and retained on the catalyst surface. Thermogravimetric analysis coupled with mass spectrometry (TGA-MS) confirmed that sulfur accumulated predominantly in the form of sulfate after the reaction. This adsorption–photocatalysis synergistic strategy effectively eliminated secondary SO_2_ emissions and mitigated catalyst deactivation caused by sulfur accumulation, thereby offering a promising approach for sustainable H_2_S abatement [109].

Wang et al. synthesized a Ti-Cr-MCM-48 photocatalyst via a one-step room-temperature co-assembly strategy, whereby Ti and Cr species were simultaneously incorporated into the framework of the mesoporous MCM-48 molecular sieve. The resulting material preserved the three-dimensional ordered mesoporous architecture of MCM-48, with Ti species highly dispersed in tetrahedral coordination and Cr predominantly present as Cr^6+^. Under visible-light irradiation, Ti-Cr-MCM-48 achieved a H_2_S removal efficiency of 92% for an inlet concentration of 35 ppm within 60 min, significantly outperforming both Ti-MCM-48, which exhibited negligible photocatalytic activity, and Cr-MCM-48. Notably, no gaseous SO_2_ byproducts were detected throughout the reaction. Despite its high initial activity, the photocatalyst underwent rapid deactivation and became nearly inactive after approximately 2 h of operation. This performance decline was primarily attributed to the accumulation of sulfate species on the catalyst surface, which blocked the mesoporous channels and covered active sites, thereby impeding mass transfer and suppressing photocatalytic reactions [110].

In another study, Chen and Xie developed a dielectric barrier discharge (DBD)-assisted photocatalytic system by integrating a TiO_2_-coated sintered metal fiber (SMF) electrode with an activated carbon fiber (ACF) lining in a DBD reactor. In this hybrid configuration, the DBD plasma generated energetic electrons and reactive oxidizing species, including •O, •OH, and O_3_. Simultaneously, ACF served as an adsorptive medium for H_2_S, enriching the pollutant near the reaction zone and prolonging its residence time, while also promoting the adsorption and decomposition of excess ozone. In parallel, ultraviolet radiation emitted from the plasma activated TiO_2_, generating photogenerated charge carriers that further enhanced oxidative degradation. The synergistic effects of plasma oxidation, adsorption enrichment, and photocatalytic oxidation enabled the system to achieve an H_2_S removal efficiency of 99.9% at an applied voltage of 25 kV and a gas residence time of 2 s, substantially outperforming stand-alone DBD treatment (39%) and ACF adsorption alone (32%) [111].

Vu et al. fabricated composite photocatalysts composed of multi-walled carbon nanotubes (MWNTs) and titanate nanotubes (TNTs) for the photocatalytic oxidation of H_2_S under UV irradiation. PL spectra showed a marked reduction in the emission intensity of TNTs after incorporation of MWNTs, indicating efficient transfer of photogenerated electrons from TNTs to the highly conductive MWNT network and, consequently, suppressed electron–hole recombination. More importantly, the hydrophobic surface of MWNTs promoted the preferential deposition of elemental sulfur on the carbon nanotubes rather than on the active TNT surface. As a result, the MWNTs/TNTs composite with a 1:1 mass ratio maintained complete H_2_S conversion for over 1000 min, while preserving 100% sulfur selectivity during the first 200 min of operation. In contrast, the sulfur selectivity of pristine TNTs declined sharply after only 30 min of reaction [112]. These results demonstrate that carbon-based conductive supports can simultaneously enhance charge-carrier separation and regulate sulfur deposition, thereby significantly improving both photocatalytic activity and long-term stability in H_2_S oxidation systems.

Wang et al. introduced pyridine moieties into a covalent polymer framework (BTC-Py) via a Schiff-base condensation strategy, achieving highly efficient and selective photocatalytic oxidation of H_2_S under visible-light irradiation. The BTC-Py photocatalyst reached an H_2_S conversion of 95.1% with nearly 100% sulfur selectivity. The pyridine units served dual functions: as Lewis basic sites, they strengthened chemisorption of acidic H_2_S molecules, and as part of a donor–acceptor (D-A) electronic structure with adjacent phenyl groups, they narrowed the band gap from 2.48 eV to 2.28 eV. Consequently, PL intensity decreased significantly, and the charge-carrier lifetime increased from 0.508 to 0.831 ns, indicating enhanced charge separation and suppressed electron–hole recombination. This study highlights the effectiveness of incorporating nitrogen-containing basic functionalities into photocatalysts to improve H_2_S adsorption, activation, and overall photocatalytic performance [113].

Similarly, Zeng et al. demonstrated that Cu-N coordination structures formed between residual Cu species and pyridine nitrogen atoms in pyridine-based conjugated polymers can significantly enhance H_2_S adsorption and activation. XPS and X-ray absorption fine structure (XAFS) analyses revealed that Cu was present in mixed Cu^+^/Cu^2+^ oxidation states and coordinated with approximately three pyridine nitrogen atoms (coordination number of about 2.8). Comparison of BPy-1 containing 1.42 wt% Cu with the Cu-depleted counterpart BPy-1-PT containing only 0.18 wt% Cu showed a dramatic decrease in H_2_S conversion from 92% to 18% following Cu removal. Furthermore, in situ Raman and DRIFTS revealed weakening of the Cu-N coordination bonds and the concurrent formation of Cu-S bonds upon H_2_S exposure. Characteristic N-H vibrational bands also emerged, indicating dissociative adsorption of H_2_S at Cu sites, where sulfur coordinated with Cu while hydrogen was transferred to adjacent pyridine nitrogen atoms. These results demonstrate that Cu-N coordination sites serve as highly effective centers for H_2_S capture and activation, while strong metal–support interactions facilitate charge transfer and thereby enhance photocatalytic oxidation performance [114].

Ren et al. synthesized a mixed-phase graphitic carbon nitride photocatalyst (HTCN) composed of triazine (PTI) and heptazine (PHI) domains via a one-step molten-salt method by tuning the precursor composition. When the PTI and PHI phases were present in an approximately 1:1 ratio, lattice mismatch-induced structural distortion disrupted the planar framework symmetry and generated an internal polarization electric field at the phase interface. This interfacial effect activated otherwise forbidden n→π* electronic transitions and extended the optical absorption edge from 460 to 700 nm. Unlike conventional band-engineering strategies based on elemental doping or defect creation, this phase-engineering approach modulated the electronic structure without introducing impurity states or defect-induced recombination centers, thereby enhancing charge-carrier separation and photocatalytic performance [115].

Wang et al. developed a CoP/CN composite photocatalyst by loading cobalt phosphide (CoP) nanoparticles onto graphitic carbon nitride (CN) for substantial visible-light-driven oxidation of low-concentration H_2_S [116]. During 35 h of continuous operation, the H_2_S conversion decreased only slightly from 94.7% to 82.4%, indicating a much lower deactivation rate than conventional TiO_2_-based systems. XPS analysis showed that the gradual activity decline was mainly due to the accumulation of small amounts of sulfate species on the catalyst surface rather than structural degradation of the CoP cocatalyst. These sulfate deposits could be effectively removed by ultrasonic washing in dilute ammonia solution, leading to substantial recovery of catalytic activity. Moreover, the intrinsic sulfur tolerance of CoP suppressed the formation of CoS and CoSO_4_, highlighting sulfur-resistant cocatalyst design as a promising strategy for improving long-term stability in H_2_S photocatalytic oxidation. Mechanistic studies further confirmed that superoxide radicals (•O^2−^) are the primary reactive species responsible for H_2_S oxidation over CoP/CN, while photogenerated holes play no direct role in the reaction.

Xu et al. provided a theoretical explanation for the CoP/CN photocatalytic system in H_2_S oxidation using DFT calculations. The valence band potential of CN (~1.6 V vs. NHE) is too low to oxidize water into hydroxyl radicals (•OH), rendering molecular oxygen activation the primary oxidation pathway. As a cocatalyst, CoP effectively adsorbs and activates O_2_ molecules, enabling their reduction by photogenerated electrons to form •O^2−^ radicals. Additionally, hydrogen-bond-assisted O_2_ activation via a quasi-Mars-van Krevelen (MvK) mechanism significantly lowers the activation barrier for O_2_ conversion. These theoretical insights is well consistent with experimental results, collectively confirming that H_2_S oxidation over CoP/CN is dominated by •O^2−^ radicals [117].

Although a wide variety of photocatalysts have been developed for H_2_S removal, their practical applicability depends not only on initial removal efficiency but also on light-harvesting capability, sulfur tolerance, regeneration ability, synthesis complexity, long-term stability, and scalability. Pristine TiO_2_, particularly commercial P25, remains the benchmark photocatalyst because of its low cost, excellent chemical stability, and well-established large-scale production, despite its limited visible-light response and rapid charge-carrier recombination. Metal-doped and noble-metal-modified TiO_2_ photocatalysts can effectively improve charge separation and broaden the light absorption range. However, these modifications often increase synthesis complexity and cost, while excessive dopant loading may introduce charge-recombination centers, compromise structural stability, or reduce long-term catalytic performance. Heterojunction photocatalysts, including Type-II, Z-scheme, and S-scheme systems, generally exhibit superior photocatalytic performance owing to more efficient interfacial charge transfer and enhanced redox capability. Nevertheless, their durability under prolonged H_2_S oxidation remains inadequately understood, particularly with respect to sulfur accumulation, interfacial degradation, and structural stability under realistic operating conditions. Emerging photocatalysts, such as metal–organic frameworks (MOFs), conjugated polymers, and semiconductor quantum dots, offer considerable potential for visible-light-driven H_2_S oxidation because of their tunable crystal structures and electronic properties. However, their widespread implementation is still constrained by complex synthesis routes, limited mechanical and chemical stability, and the lack of scalable, cost-effective manufacturing technologies.

This section summarizes the reported studies on the photocatalytic oxidation of H_2_S, as compiled in Table 1 and Table 2. As evident from these tables, the experimental conditions vary considerably among the reported studies, making direct performance comparisons challenging. To enable a systematic evaluation, several key parameters were extracted and compared, including operating conditions (temperature, relative humidity, and reaction scale), light source and irradiance, initial H_2_S concentration, reactor configuration, reaction time, photocatalyst composition and dosage, and H_2_S removal or conversion efficiency. It should be noted that some operating parameters were not explicitly reported in the original publications and were therefore estimated or calculated from the available information where possible. Despite the variability in experimental conditions, the compiled data provide a comprehensive overview of the performance of different photocatalysts and reaction systems, offering valuable insights into the current state of photocatalytic H_2_S oxidation and the factors governing catalyst performance.

## 3. Engineering Challenges, Catalyst Stability and Practical Implementation

The engineering performance of photocatalytic reactors is governed by reactor configuration, light distribution, mass transfer, and hydrodynamic characteristics. Consequently, reactor design should maximize photon utilization and catalyst accessibility while minimizing mass-transfer limitations and energy consumption during long-term operation.

Representative reactor designs have employed different engineering strategies to enhance photocatalytic performance. An internally circulating photocatalytic reactor equipped with a 185 nm ozone-generating UV lamp and TiO_2_ immobilized on nickel foam improved gas–solid contact through forced gas recirculation, resulting in more uniform reactant distribution over the catalyst surface [79]. In contrast, a dielectric barrier discharge (DBD)-assisted photocatalytic reactor integrated TiO_2_-coated sintered metal fibers with activated carbon fiber (ACF), enabling in situ activation of the photocatalyst by plasma-generated UV irradiation while simultaneously enriching H_2_S near the catalyst surface through adsorption by the ACF layer [111]. Under a peak voltage of 25 kV and a gas residence time of 2 s, this integrated system achieved an H_2_S removal efficiency of 99.9% for an inlet concentration of 75 mg m^−3^, highlighting the strong synergistic effects of plasma activation, adsorption, and photocatalysis.

Light penetration remains a major challenge for the scale-up of photocatalytic reactors. Although the reflective inner wall of the internally circulating reactor improved photon utilization, it also underscored the rapid attenuation of UV irradiation within photocatalytic systems. Comparative studies using different irradiation wavelengths showed that a 185 nm UV source achieved an H_2_S removal efficiency of 97.4% after 3 h, whereas 254 and 365 nm irradiation resulted in efficiencies of only 39.1% and 33.1%, respectively [79]. In addition to its higher photon energy, the 185 nm source generated substantially higher ozone concentrations (5.95 ppm), which promoted hydroxyl radical formation and suppressed electron–hole recombination. However, short-wavelength UV light is also more readily absorbed by gas molecules and the outer catalyst layer, thereby reducing the effective light penetration depth. Consequently, optimizing catalyst coating thickness, reactor geometry, and light-source configuration is essential for maintaining efficient photon transport and catalytic performance during reactor scale-up.

Pressure drop is another critical engineering consideration, particularly in packed-bed and hybrid photocatalytic reactors. Although incorporating ACF significantly enhances H_2_S adsorption and prolongs gas residence time, it inevitably increases flow resistance. During prolonged operation, saturation of adsorption sites and inadequate adsorbent regeneration further exacerbate pressure drop, leading to higher energy consumption and reduced operational stability. Therefore, catalyst loading, adsorbent packing density, and regeneration strategies should be jointly optimized to achieve an appropriate balance between H_2_S removal efficiency and operating costs [84].

Operating conditions also exert a profound influence on reactor performance. Moderate relative humidity promotes hydroxyl radical (•OH) generation and enhances H_2_S oxidation, whereas excessive moisture suppresses photocatalytic activity through competitive adsorption on catalyst surfaces and quenching of plasma-generated reactive species. Similarly, an oxygen concentration close to that of ambient air (~21 vol%) provides optimal degradation performance by facilitating ROS generation without excessively competing with H_2_S for active adsorption sites. Increasing the gas residence time further improves H_2_S conversion by prolonging gas–solid contact and increasing the probability of surface reactions; however, these benefits are accompanied by lower throughput, higher pressure drop, and increased energy demand. On the whole, these observations demonstrate that the engineering optimization of photocatalytic reactors requires careful coordination of reaction kinetics, photon transport, mass transfer, hydrodynamic resistance, and energy efficiency to achieve stable and economically viable operation.

During photocatalytic H_2_S oxidation, catalyst deactivation primarily results from the accumulation of sulfur-containing intermediates on the catalyst surface. Among these species, elemental sulfur (S^0^) is widely regarded as the principal cause of TiO_2_ deactivation because of its low reactivity toward further oxidation and its strong tendency to cover catalytically active sites. The deposition of S^0^ not only blocks the adsorption and activation of H_2_S molecules but also impedes the interfacial transfer of photogenerated charge carriers, thereby suppressing the generation of ROS and progressively diminishing photocatalytic activity. X-ray photoelectron spectroscopy (XPS) analyses have shown that the surface S^0^/SO_4_^2−^ ratio increases markedly after catalyst deactivation, indicating that the accumulation of incompletely oxidized sulfur species is a characteristic indicator of catalyst poisoning [101].

Catalyst regeneration is fundamentally based on the removal or further oxidation of deposited sulfur species to restore accessible active sites. Li et al. demonstrated that TiO_2_ could be regenerated in situ using a 185 nm O_3_/UV system, in which ozone generated under UV irradiation, together with the associated ROS, oxidized deposited S^0^ into SO_4_^2−^ [79]. This process effectively regenerated surface-active sites and restored photocatalytic activity. Compared with conventional thermal regeneration, the O_3_/UV strategy eliminates the need for external heating and can be directly integrated into the photocatalytic process, making it particularly attractive for continuous operation [94]. Furthermore, SO_4_^2−^ is thermodynamically more stable than S^0^, and its formation suppresses the continuous accumulation of S^0^ deposits on the catalyst surface. Surface sulfate species may also modify the acidity of TiO_2_, which can contribute to improved long-term catalyst stability.

Current studies indicate that catalyst deactivation during photocatalytic H_2_S oxidation can generally be classified into three categories: (i) surface deposition of S^0^, which represents the predominant and largely reversible deactivation pathway; (ii) excessive accumulation of SO_4_^2−^ species, which may gradually block pores and reduce the accessible surface area during prolonged operation; and (iii) photocorrosion or structural degradation, which is relatively insignificant for TiO_2_ but can become severe in less stable photocatalysts, such as CdS [108]. Among these mechanisms, sulfur deposition remains the primary cause of reversible deactivation, highlighting the importance of in situ oxidation of sulfur species as the most effective regeneration strategy.

At present, pristine TiO_2_, particularly commercial P25, remains the most promising photocatalyst for practical applications because of its low cost, excellent chemical stability, well-established large-scale production, and reliable photocatalytic performance. Although modified photocatalysts, including metal-doped TiO_2_, heterojunctions, and MOF-based materials, generally exhibit superior H_2_S removal efficiencies under laboratory conditions, their practical deployment is still constrained by higher fabrication costs, more complex synthesis procedures, and insufficient evidence regarding long-term durability and operational reliability under realistic conditions. In practical environments, such as livestock facilities and wastewater treatment plants, catalyst performance may be substantially deteriorated by dust deposition, humidity fluctuations, and competitive adsorption from multicomponent gas mixtures, thereby diminishing the performance advantages of modified photocatalysts over pristine TiO_2_ and reducing their economic competitiveness [5].

The selection of the light source also has a profound influence on reactor economics and overall process sustainability. Although 185 nm ozone-generating UV lamps provide excellent H_2_S removal performance, their widespread application is limited by relatively high energy consumption, short service lifetimes, and the environmental concerns associated with mercury-containing lamps. By comparison, UV-LEDs offer lower power consumption, longer operational lifetimes, rapid start-up, and improved environmental compatibility. As their wall-plug efficiency and output power continue to improve, UV-LEDs are expected to become an increasingly attractive option for photocatalytic H_2_S treatment from both life-cycle and economic perspectives [101]. Furthermore, integrating non-thermal plasma with photocatalysis not only enhances H_2_S oxidation through the generation of additional reactive species but also enables in situ catalyst regeneration, thereby extending catalyst lifetime and reducing long-term operating costs. Consequently, commercial TiO_2_ currently offers the most favorable balance among performance, cost, durability, and engineering feasibility, whereas advanced composite photocatalysts are likely to become economically viable only if their superior activity can offset the additional material and fabrication costs or if their service life can be substantially prolonged through efficient in situ regeneration and optimized reactor design.

Taken together, the practical implementation of photocatalytic H_2_S oxidation depends not only on the intrinsic activity of photocatalysts but also on reactor engineering, catalyst durability, and process economics. Future research should therefore adopt an integrated design strategy that simultaneously optimizes photocatalyst composition, reactor geometry, light-source configuration, gas–solid mass transfer, and regeneration protocols. Particular emphasis should be placed on maximizing photon utilization, minimizing hydraulic resistance and energy consumption, and suppressing sulfur-induced deactivation through continuous in situ regeneration. Such a multidisciplinary approach is expected to enhance catalyst stability, improve overall process efficiency, and accelerate the scale-up and commercialization of photocatalytic H_2_S removal technologies.

## 4. Mechanisms of Photocatalytic Oxidation for H_2_S Removal

To date, substantial efforts have been devoted to elucidating the mechanisms of photocatalytic H_2_S oxidation over both TiO_2_-based and non-TiO_2_-based photocatalysts, with TiO_2_-based systems being the most extensively investigated. It is generally accepted that photocatalytic H_2_S oxidation involves four key stages: adsorption, activation, intermediate transformation, and deep oxidation. The reaction pathways and product distribution are determined by the interplay among ROS, catalyst surface properties, and operating conditions.

Adsorption of H_2_S onto the catalyst surface is a prerequisite for subsequent photocatalytic reactions. Previous studies have shown that O-Ti^4+^ sites on TiO_2_ serve as the primary active centers for H_2_S adsorption and activation. H_2_S molecules preferentially adsorb through coordination of the sulfur atom with surface Ti^4+^ sites rather than through interactions with surface hydroxyl groups (−OH) [71,87]. Only after saturation of the O-Ti^4+^ sites does adsorption on surface hydroxyl groups become outstanding. After adsorption, H_2_S can be activated by photogenerated holes (h^+^) or OH, generating the key hydrosulfide radical intermediate (•HS) [71], as described by Equations (1) and (2):

(1) $${H_{2}S}_{ads}+h^{+}\to {{•H}_{2}S}_{ads}^{+}↔{•HS}_{ads}+H^{+}$$

(2) $${H_{2}S}_{ads}+{•OH}_{ads}\to {•HS}_{ads}+{H_{2}O}_{ads}$$

Notably, the oxidation potential of sulfide species, including H_2_S (approximately +1.8 V vs. NHE), is significantly lower than both the valence-band potential of TiO_2_ (approximately +3.0 V vs. NHE) and the redox potential required for •OH generation (+2.59 to +2.74 V vs. NHE). Therefore, H_2_S can be directly oxidized by photogenerated holes without exclusively relying on the indirect oxidation pathway mediated by •OH radicals. This feature makes H_2_S an important model compound for investigating direct hole-driven oxidation processes in photocatalytic systems [36].

Once •SH radicals are generated, their subsequent transformation is primarily controlled by oxygen availability, ROS composition, and the overall oxidation capacity of the photocatalytic system. Accordingly, two representative reaction pathways can be distinguished.

(i) Sulfate (SO_4_^2−^) formation pathway: deep oxidation process.

Under oxygen-rich conditions, •SH radicals can further react with adsorbed oxygen (O_2,ads_) or superoxide radicals (•O_2_^−^) to form •OOSH intermediates, which are subsequently transformed into adsorbed SO_2_ species. The generated SO_2_ is further oxidized to SO_3_ and ultimately hydrated to yield surface-bound sulfate species (H_2_SO_4_/SO_4_^2−^) [34,71] (Figure 6a,b). The overall process can be expressed by Equations (3)–(6):

(3) $${•HS}_{ads}+O_{2,ads}\to {•OOSH}_{ads}$$

(4) $${•OOSH}_{ads}+O_{2,ads}\to {SO}_{2,ads}+{•OH}_{ads}$$

(5) $${SO}_{2,ads}+\frac{1}{2}O_{2,ads}\to {SO}_{3,ads}$$

(6) $${SO}_{3,ads}+{H_{2}O}_{ads}\to {H_{2}SO}_{4,ads}$$

In addition, under strongly oxidative conditions, H_2_S can undergo direct eight-electron oxidation by highly reactive oxidizing species, such as •OH and H_2_O_2_, resulting in the complete conversion of S^0^ to SO_4_^2−^. This pathway bypasses part of the sequential oxidation process involving sulfur-containing intermediates and facilitates the efficient mineralization of H_2_S in highly oxidative photocatalytic system [86]. The overall reaction can be expressed as Equation (7):

(7) $$H_{2}S+8•OH\to {SO}_{4}^{2-}+2H^{+}+4H_{2}O$$

Sulfate is generally considered the predominant final product of H_2_S photocatalytic oxidation and can be readily detected using surface characterization techniques such as XPS and FTIR. However, the progressive accumulation of sulfate on the catalyst surface gradually blocks active sites and impedes the adsorption of reactant molecules, ultimately leading to catalyst deactivation and a decline in photocatalytic oxidation performance.

(ii) Formation and reoxidation pathway of elemental sulfur (S^0^): incomplete oxidation process.

Under conditions of limited oxygen availability or insufficient oxidative capacity, •HS can undergo radical coupling or partial oxidation to yield elemental sulfur (S^0^), as described by Equations (8) and (9) [79]:

(8) $$H_{2}S+2•OH\overset{incomplete\;oxidation}{\to }S↓+2H_{2}O$$

(9) $$H_{2}S+H_{2}O_{2}\overset{incomplete\;oxidation}{\to }S↓+2H_{2}O$$

The generated elemental sulfur acts not only as an important reaction intermediate but also as a key factor in catalyst deactivation. It can subsequently undergo further oxidation to form sulfate species, as described by Equation (10):

(10) $$S+\frac{3}{2}O_{2}+H_{2}O\to {H_{2}SO}_{4}$$

Previous studies have shown that elemental sulfur (S^0^) accumulates rapidly on the catalyst surface during the initial stage of the reaction. As the reaction proceeds, a portion of the deposited S^0^ is gradually oxidized to SO_4_^2−^, leading to a progressive decline in its surface concentration [118]. Therefore, enhancing the oxidative capacity of the reaction system, for example by increasing O_2_ concentration or introducing O_3_, can promote the deep oxidation of S^0^ and consequently mitigate catalyst deactivation.

(iii) Formation and release of SO_2_ as a gaseous intermediate.

Under conditions such as elevated temperatures, low humidity, or severe catalyst deactivation, adsorbed SO_2_ species may desorb from the catalyst surface and be released into the gas phase, resulting in the formation of SO_2_ as an undesirable by-product. Previous studies have demonstrated that sulfate species accumulated on the catalyst surface can react with photogenerated holes during prolonged operation to generate sulfate radicals (•SO_4_^−^) [95] (Figure 6b), as described by Equation (11):

(11) $${SO}_{4}^{2-}+h^{+}\to {•SO}_{4}^{-}$$

The sulfate radical (•SO_4_^−^) exhibits a very high oxidation potential (around +2.6 V vs. NHE) and can further drive the oxidation of H_2_S to SO_2_, thereby substantially enhancing SO_2_ selectivity. In severely deactivated systems, SO_2_ can even become the predominant sulfur-containing product, with selectivity approaching 100% [71].

In vacuum ultraviolet photocatalytic oxidation (VUV-PCO) systems, irradiation at 185 nm can directly photolyze molecular oxygen to generate O_3_, which subsequently decomposes into highly reactive species, such as O(^1^D) and •OH, thereby enhancing H_2_S photocatalytic oxidation. In addition, O_3_ can directly react with H_2_S to produce SO_2_, as described by Equation (12):

(12) $$H_{2}S+O_{3}\to {SO}_{2}+H_{2}O$$

In addition to SO_2_ and S^0^, various oxygen-containing sulfur intermediates have been identified during the photocatalytic oxidation of H_2_S. Using in situ FTIR spectroscopy, Kataoka et al. observed weak absorption bands at 1093 and 996 cm^−1^ on TiO_2_ thin films during H_2_S photocatalytic oxidation, which were assigned to sulfite radical species (•SO_3_^−^). The intensities of these bands increased during the intermediate stage of the reaction and subsequently decreased, suggesting that •SO_3_^−^ serves as a key transient intermediate in the oxidation pathway from H_2_S to SO_4_^2−^ [86]. Furthermore, Canela et al. detected additional sulfur oxyanion intermediates, including dithionate (S_2_O_6_^2−^, 1215 cm^−1^) and metabisulfite (S_2_O_5_^2−^, 1052 cm^−1^), providing further evidence that H_2_S photocatalytic oxidation proceeds via a complex, multistep reaction network rather than a direct one-step conversion [68].

Recent studies have demonstrated that certain photocatalysts, such as In-TiO_2_ and B-TiO_2_/LDH, exhibit pronounced photothermal synergistic effects during H_2_S oxidation (Figure 4). Even in the absence of light, these catalysts are capable of converting H_2_S into H_2_O and S^0^ via thermocatalytic pathways. Upon illumination, the generated S^0^ can be further oxidized to SO_4_^2−^, thereby enhancing overall sulfur mineralization [72,95]. Moreover, light on–off–on cycling experiments have shown that catalysts pre-illuminated under irradiation retain remarkable activity during subsequent dark periods. This behavior indicates the formation of persistent surface oxidative species, such as SO_4_^2−^ and •SO_4_^−^, which continue to participate in H_2_S oxidation even after the light source is removed [95]. These observations highlight the importance of integrating photochemical and thermochemical processes to improve both catalyst durability and sulfur oxidation efficiency.

Humidity exerts a pronounced dual effect on the photocatalytic oxidation of H_2_S. On one hand, water molecules serve as a key source of •OH radicals, which facilitate the oxidative degradation of H_2_S. On the other hand, excessive humidity can negatively impact the reaction by competing with H_2_S for Ti^4+^ active sites and forming a water film on the catalyst surface, thereby increasing mass-transfer resistance and hindering reactant diffusion. Alonso-Tellez et al. reported that H_2_S removal was most effective under dry conditions in a UV-A (365 nm)/TiO_2_ system. As relative humidity increased from 0% to 100%, H_2_S conversion decreased from 56% to 20%, while SO_2_ selectivity rose from 14% to 50% [71], indicating that excessive moisture suppresses complete oxidation to sulfate and promotes SO_2_ release. In contrast, VUV/O_3_-assisted systems operating at 185 nm benefit from continuous ozone photolysis, which generates abundant •OH radicals, yielding an optimal relative humidity of approximately 80% [17,79]. Moreover, although elevated temperatures generally accelerate reaction kinetics, they simultaneously weaken the adsorption of both H_2_S and SO_2_ on catalyst surfaces, increasing the likelihood of SO_2_ desorption into the gas phase [71].

The electronic band structure of photocatalysts plays a pivotal role in determining the reaction pathway of H_2_S oxidation. For instance, In^3+^ doping shifts the CB edge of TiO_2_ from −0.29 to −0.50 eV, rendering it more negative than the reduction potential of O_2_/•O^2−^ (−0.33 eV). This band-structure modulation enables the activation of molecular oxygen and the generation of •O^2−^, whereas pristine TiO_2_ predominantly produces •OH radicals. In addition, In^3+^ doping substantially decreases the oxygen-vacancy formation energy from +5.43 to −1.28 eV, thereby facilitating the formation of oxygen vacancies and enhancing H_2_S adsorption. Consequently, the adsorption energy of H_2_S increases markedly from −0.04 to −4.07 eV, accompanied by an elongation of the S-H bond length from 1.35 to 1.64 Å, indicating pronounced activation of the H_2_S molecule and weakening of the S-H bond [72]. Similar effects have been observed in CoP/CN heterojunction systems, where CoP serves as an electron extraction and accumulation center, promoting electron transfer to adsorbed O_2_ and thereby enhancing oxygen activation. The adsorption energy of O_2_ on CoP/CN reaches −1.74 eV, favoring the formation of •O^2−^ rather than •OH radicals. Furthermore, the catalyst predominantly follows a two-electron oxygen reduction pathway (n = 2.21), which facilitates efficient generation of reactive oxygen species and contributes to its superior photocatalytic performance in H_2_S oxidation [116].

In summary, the fundamental mechanism of photocatalytic H_2_S oxidation can be described as follows: upon adsorption onto the catalyst surface, H_2_S is activated by photogenerated holes (h^+^) or •OH, forming •SH radicals. These •SH intermediates then undergo further oxidation, radical coupling, or deep mineralization reactions, ultimately yielding S^0^, SO_2_, and SO_4_^2−^. Among these species, •SH serves as the key intermediate connecting multiple reaction pathways, and its subsequent transformation largely determines product selectivity and catalyst stability. The principal reactive species involved include •OH, •O^2−^, •SH, and •SO_4_^−^, whereas important reaction intermediates comprise •OOSH, •SO_3_^−^, S_2_O_6_^2−^, S_2_O_5_^2−^, SO_2_, and S^0^. Under varying reaction conditions, the distribution of products and overall photocatalytic performance are synergistically governed by factors such as oxygen availability, humidity, temperature, catalyst band structure, and surface properties.

## 5. Conclusions and Future Perspectives

Overall, photocatalytic oxidation has emerged as a green, efficient, and promising technology for H_2_S removal, particularly from low-concentration and dispersed emission sources. Significant advances have been achieved over the past decade. Extensive research efforts have focused on the development of TiO_2_-based photocatalysts through crystal-phase engineering, elemental doping, defect engineering, and heterojunction construction [119]. In parallel, emerging photocatalytic materials, including MOFs, g-C_3_N_4_, conjugated polymers, and carbon-based composites, have demonstrated considerable potential for H_2_S abatement. Despite these advances, current research remains dominated by TiO_2_-based systems, whereas investigations of alternative visible-light-responsive photocatalysts are still relatively limited. Moreover, the underlying reaction mechanisms, interfacial charge-transfer processes, and structure–activity relationships of these emerging materials remain insufficiently understood. Conventional photocatalysts also continue to suffer from intrinsic limitations, including narrow light-response ranges, rapid recombination of photogenerated charge carriers, and low solar energy utilization efficiency, resulting in photocatalytic performance and long-term stability that are often inadequate for practical industrial applications.

A major challenge in H_2_S photocatalytic oxidation is the accumulation of sulfur-containing oxidation products, particularly S^0^ and SO_4_^2−^, on catalyst surfaces. These deposits progressively block active sites and exacerbate light-shielding effects, ultimately causing catalyst deactivation [120]. Under oxygen-rich conditions, H_2_S is preferentially converted to sulfate, which minimizes the emission of gaseous by-products such as SO_2_. However, excessive sulfate accumulation increases regeneration requirements and adversely affects long-term operational stability. Conversely, under oxygen-deficient conditions or when the oxidation capability of the catalyst is insufficient, H_2_S tends to undergo partial oxidation, producing elemental sulfur and polysulfide intermediates that can likewise induce catalyst poisoning and activity loss. Therefore, simultaneously achieving substantial H_2_S conversion, desirable product selectivity, and resistance to sulfur-induced deactivation remains a central challenge in the development of practical photocatalytic oxidation systems.

To address these limitations, various multi-field coupling strategies, including photothermal catalysis, ozone-assisted photocatalytic oxidation, and plasma-assisted processes, have been explored to enhance solar energy utilization and promote the ROS generation [121]. Among these approaches, photothermal catalysis is particularly attractive because localized thermal effects generated under light irradiation can accelerate surface reaction kinetics while facilitating charge-carrier migration and separation [122]. As a result, photothermal systems often exhibit superior H_2_S removal efficiency and operational stability compared with conventional photocatalytic processes. Nevertheless, previous studies have shown that sulfate and elemental sulfur deposition cannot be completely eliminated, even in synergistic catalytic systems, and catalyst deactivation remains evident during prolonged operation [123,124]. These observations suggest that simply enhancing oxidation capability is insufficient to fundamentally overcome the stability limitations of photocatalytic H_2_S oxidation.

Looking ahead, overcoming the current limitations of photocatalytic H_2_S oxidation will require the synergistic advancement of both photocatalyst design and reactor engineering. From a materials perspective, future research should prioritize the development of photocatalysts with enhanced visible-light harvesting, efficient charge separation, superior sulfur tolerance, and long-term structural stability. Rational design strategies, including S-scheme and Z-scheme heterojunction engineering, defect engineering, hierarchical architectures, and the integration of emerging material platforms such as MOFs, conjugated polymers, metal phosphides, single-atom catalysts, and semiconductor quantum dots, are expected to further improve photocatalytic activity, selectivity, and durability. From an engineering perspective, greater emphasis should be placed on optimizing reactor geometry, light-field distribution, catalyst immobilization, gas–solid mass transfer, and in situ regeneration strategies to maximize photon utilization, minimize mass-transfer resistance and pressure drop, and ensure stable long-term operation under practical conditions. Meanwhile, the integration of advanced in situ and operando characterization techniques with multiscale theoretical simulations, including DFT calculations, will provide deeper insight into the adsorption, activation, and transformation of H_2_S and sulfur-containing intermediates, thereby establishing mechanistic principles for the rational design of photocatalysts and photocatalytic reactors. Ultimately, the integration of advanced photocatalyst development with reactor engineering, mechanistic understanding, and process optimization will be essential for overcoming the remaining challenges of photocatalytic H_2_S oxidation. Such a multidisciplinary approach is expected to accelerate the translation of photocatalytic H_2_S removal from laboratory-scale demonstrations to scalable, energy-efficient, and economically viable technologies for practical environmental applications.

## Figures and Tables

**Table 1:** Summary of photocatalytic methods for H_2_S removal using TiO_2_-based photocatalysts.

Experimental Conditions Temp/RH	Year	Light Source	Irradiance (mW/cm^2^)	H_2_S Concentration	Reactor or Reaction Place	Treatment Tim	Photocatalyst Dose	Conversion	Reference
22–160 °C/Dry	2012	UV-A Blacklight (380 nm)	3.3	15 ppm	single pass annular Pyrex reactor	5 h	TiO_2_ P25 (0.04 mg/cm^2^)	50%	[71]
							TiO_2_ P25 (0.44–1.77 mg/cm^2^)	100%	
25 °C/Dry	2024	UV light (200–400 nm)	NR	100 ppm	fixed-bed quartz tubular reactor	2 h	TiO_2_	7.1%	[72]
							P25 TiO_2_	32.2%	
							Al-TiO_2_	73.2%	
							Ga-TiO_2_	82.1%	
							In-TiO_2_	100%	
Dry	2014	UV-A Blacklight (365 nm)	3.3	15 ppm	Annular Pyrex reactor	20 h	5% WO_3_/TiO_2_ UV100 (0.21 mg/cm^2^)	80%	[73]
							10% WO_3_/TiO_2_ UV100 (0.21 mg/cm^2^)	75%	
							15% WO_3_/TiO_2_ UV100 (0.21 mg/cm^2^)	40%	
							20% WO_3_/TiO_2_ UV100 (0.21 mg/cm^2^)	53%	
Dry	2008	UV-A (365 nm)	NR	15 ppmv	Continuous flow flat reactor	10 h	SG-CA (70 μg/cm^2^)	30%	[75]
							SG-PET (110 μg/cm^2^)	20%	
							SG-G (130 μg/cm^2^)	20%	
							SG-G350 (110 μg/cm^2^)	10%	
20 °C	2014	UV-A and VIS	9.0 (UV-A) + 6.0 (VIS)	2000 ppm	Quartz tube reactor	400 min	TiO_2_ powder	NR	[76]
							TiO_2_/support under dry conditions		
							TiO_2_/support with water		
							without any photocatalyst		
RT/Wet	2017	UV light	NR	200 ppm	batch reactor	60 min	TiO_2_@MIL-101	90.1%	[78]
							Hollow TiO_2_	68.8%	
							Bulk TiO_2_	42.2%	
30.0–32.8 °C/21%	2012	UV (185 nm)	NR	203.31–215.31 mg/m^3^	Photoreactor	180 min	TiO_2_	28.37%	[79]
30.0–32.8 °C/43%								38.07%	
30.0–32.8 °C/62%								73.14%	
30.0–32.8 °C/70%								78.53%	
30.0–32.8 °C/80%								97.36%	
30.0–32.8 °C/90%								96.95%	
RT/50%	2005	UV	2.0	0.75 ppm	Stainless steel reactor	12 h	Ag-PCF (1.0)	80%	[80]
							Ag-PCF (0.2)	70%	
33–50 °C/25%	2007	UV-A	NR	35 ppm	Annular reactor	14 h	PET-RT	33%	[82]
							CA-RT	20%	
							glass Raschig ring	25%	
25 °C/18–24%	2022	UV (254 nm)	18	10 ppm	Plug flow quartz reactor	3 h	P25	70%	[83]
							TiO_2_-350 °C	80%	
							TiO_2_-450 °C	100%	
							TiO_2_-700 °C	70%	
NR/40%	2016	UV-A (365 nm)	5	1000 ppm	Tubular reactor	300 min	Reactor A (raw zeolite filling)	0%	[84]
							Reactor B (TiO_2_ film coating)	33%	
							Reactor C (zeolite/TiO_2_ filling)	90%	
							Reactor C (TiO_2_ film coating and zeolite/TiO_2_ filling)	98.1%	
25 °C	2015	UV-A (365 nm)	3	1000 ppm	Tubular reactor	5 h	NaOH/400 °C	93%	[85]
							NaOH	87%	
							H_2_O/400 °C	82%	
							H_2_O	72%	
RT/50%	2018	VUV (185 nm)	NR	150 ppm	Fixed-bed reactor	60 min	TiO_2_	76.5	[88]
							1 wt% Ni-TiO_2_	71.3	
							1 wt% Mn-TiO_2_	89.9	
							1 wt% Cu-TiO_2_	73.8	
							1 wt% Co-TiO_2_	76.9	
40 °C/Dry	2014	UV-A (365 nm)	3.3	15 ppm	Annular reactor	300 min	P25	27%	[91]
							TiO_2_ nanorods (350 °C)	35%	
							TiO_2_ nanorods (380 °C)	64%	
							TiO_2_ nanorods (420 °C)	55%	
							TiO_2_ nanorods (500 °C)	45%	
							TiO_2_ nanorods (600 °C)	31%	
							TiO_2_ nanorods (700 °C)	27%	
Dry	2015	UV-A (365nm)	4.53	15 ppm	Single-pass annular Pyrex reactor	3 h	N_1_TiNT-400	98%	[92]
25 °C/Dry	2024	UV (365 nm)	NR	5 ppm	NR	10 min	TiO_2_	33.5%	[93]
							0.1%-Hg/TiO_2_	54.2%	
							1%-Hg/TiO_2_	96.1%	
							2%-Hg/TiO_2_	100%	
							5%-Hg/TiO_2_	100%	

**Table 2:** Summary of photocatalytic methods for H_2_S removal using composite photocatalysts.

Experimental Conditions Temp/RH	Year	Light Source	Irradiance (mW/cm^2^)	H_2_S Concentration	Reactor or Reaction Place	Treatment Time	Photocatalyst (Dose)	Conversion	Reference
RT/Dry	2023	LED (420 nm)	50	20 ppm	Flow reaction system	600 min	SiO_2_@α-Fe_2_O_3_@COF	95.2%	[101]
							SiO_2_@COF	75.2%	
25 °C/Dry	2025	Visible light (λ > 420 nm)	NR	50–250 ppm	Tubular quartz reactor	120 min	IBP-3	92.3%	[105]
25 °C/Dry	2026	Visible light (λ > 420 nm)	NR	100 ppm	Tubular quartz reactor	8 h	In-TiO_2_-0.4@Bi_2_MoO_6_	65%	[106]
30 °C/30%	2010	UV-A (365 nm)	4.4	15 ppm	Plug flow reactor	12 h	P25 TiO_2_	>95%	[109]
							coated TiO_2_-SiMgOX	90%	
							incorporated TiO_2_-SiMgOX	30%	
							SiMgOX	5%	
30 °C/Wet	2012	Visible light	NR	35 ppm	Glass tubular reactor	2 h	Ti-Cr-MCM-48	15%	[110]
							Cr-MCM-48	5%	
							Ti-MCM-48	0%	
25 °C/0%	2013	DBD + UV	NR	75 mg/m^3^	Rector A	30 d	TiO_2_/SMF+ACF	100%	[111]
					Rector B		TiO_2_/SMF	60%	
					Rector C		Ni-Cr	40%	
					Rector D		Ni-Cr+ACF	93%	
					Rector E		ACF	0%	
Dry	2017	UV-A (350 nm)	NR	15 ppm	Annular Pyrex reactor	1100 min	1/10MWNTs/TNTs	80%	[112]
							1/1MWNTs/TNTs	100%	
							2/1MWNTs/TNTs	65%	
30 °C/Dry	2024	Visible light (420 nm LED)	8.7	20 ppm	fix bed continuous flow system	NR	BTC-B	40.8%	[113]
							BTC-Py_0.33_	53.3%	
							BTC-Py_0.66_	76.3%	
							BTC-Py	95.1%	
30 °C/Dry	2025	Visible light (420 nm LED)	NR	20 ppm	fix bed continuous flow system	NR	BPy-1	92%	[114]
							BPy-2	78%	
							BPy-3	49%	
							BPy-1-PT	18%	

## Data Availability

No new data were created or analyzed in this study. Data sharing is not applicable to this article.
